# Targeted Extracellular Vesicles Deliver Asiaticoside to Inhibit AURKB/DRP1‐Mediated Mitochondrial Fission and Attenuate Hypertrophic Scar Formation

**DOI:** 10.1002/advs.202517108

**Published:** 2026-02-04

**Authors:** Luyu Li, Chenli Si, Xue Wang, Xiaojin Wu, Ying Shang, Shengfang Ge, Yong Wang, Yong Zuo, Zhen Zhang

**Affiliations:** ^1^ Department of Dermatology Shanghai Ninth People's Hospital Shanghai Jiao Tong University School of Medicine Shanghai China; ^2^ Department of Laser and Aesthetic Medicine Shanghai Ninth People's Hospital Shanghai Jiao Tong University School of Medicine Shanghai China; ^3^ Department of Ophthalmology Shanghai Key Laboratory of Orbital Diseases and Ocular Oncology Shanghai Ninth People's Hospital Shanghai Jiao Tong University School of Medicine Shanghai China; ^4^ Department of Neurology Zhongshan Hospital Fudan University Shanghai China; ^5^ Department of Biochemistry and Molecular Cell Biology Shanghai Key Laboratory for Tumor Microenvironment and Inflammation Shanghai Jiao Tong University School of Medicine Shanghai China

**Keywords:** engineered EVs, hypertrophic scars, machine‐learning, mitochondrial fission, target delivery

## Abstract

Hypertrophic scars (HS) are fibroproliferative lesions arising from aberrant wound healing, their high incidence is countered by a lack of effective interventions owing to an incomplete understanding of pathogenesis. Here, we identify dysregulated mitochondrial dynamics as a key driver of HS and develop a new targeted therapy. Specifically, excessive mitochondrial fission was observed in macrophages derived from both human and murine HS tissues. In vitro and in vivo experiments revealed that this imbalance is governed by AURKB‐mediated phosphorylation of DRP1 at Ser616 site. Through machine‐learning coupled with biological validation, we identified the natural small‐molecule Asiaticoside (AS) as a potent AURKB inhibitor. However, AS has limited targeting accuracy and poor bioavailability. To overcome these challenges, we developed cRGD‐decorated extracellular vesicles (EVs) loaded with AS (AS@cRGD‐EVs), enabling targeted delivery of AS to macrophages within wound tissue. In vitro and in vivo studies showed that AS@cRGD‐EVs effectively restrained macrophage mitochondrial fission, rebalanced the inflammatory milieu, and conferred significant anti‐scarring efficacy in murine HS models. This work establishes mitochondrial dynamics as a therapeutic axis for HS and delivers a targeted nanotherapeutic ready for translational evaluation.

## Graphical abstract

Hypertrophic scar formation is driven by excessive mitochondrial fission in wound macrophages, which we discover is governed by a novel AURKB‐DRP1(Ser616) axis. The study develops a targeted therapy using cRGD‐decorated extracellular vesicles to deliver the natural compound Asiaticoside specifically to macrophages. This nanoplatform effectively inhibits this pathological axis, rebalances inflammation, and attenuates scar formation.

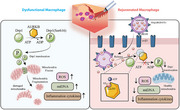

**Abbreviations**
AS: asiaticoside
AURKB: aurora kinase B
DCFH‐DA: 2',7', dichlorodihydrofluorescein diacetate
DRP1: dynamin‐related protein 1
EVs: extracellular vesicles
HS: hypertrophic scars
LPS: lipopolysaccharide
MACS: magnetic separation
MMP: mitochondrial membrane potential
MST: microscale thermophoresis
mtDNA: mitochondrial DNA
PBS: phosphate‐buffered saline
ROS: reactive oxygen species

## 1 Introduction

Hypertrophic scar (HS) is a prevalent and substantial global health concern [1], characterized by persistent local inflammation, excessive fibroblast activity, and the deposition of collagen [2, 3]. Various factors that lead to skin dermis injuries can result in HS, including trauma, burns, surgical incisions, and skin infections [4, 5]. Patients with severe HS often suffer from mobility issues, chronic pain, and discomfort with their appearance. A study found that about 15.3% of patients had psychological impacts that reduced their quality of life and delayed their reintegration into the community after injury [6]. The methods for treating HS mainly include surgical excision, intralesional steroid therapy, laser treatment [7]. However, these treatments often fail to provide long‐term solutions. Surgical excision, although effective in removing the scar tissue, is frequently associated with high recurrence rates [8, 9], as the underlying pathophysiological mechanisms that drive scar formation remain unchanged. Similarly, intralesional steroid therapy, while reducing inflammation and scar size, often leads to only temporary improvements and can cause local skin atrophy, thinning, rupture, capillary dilation and other new skin problems [10]. These challenges highlight the need for new approaches to effectively prevent scar formation and recurrence, emphasizing the importance of promptly implementing preventive measures following wound formation.

HS formation is a multi‐stage process that involves three distinct yet interconnected phases: inflammation, proliferation, and remodeling [2]. Each phase is driven by unique molecular and cellular mechanisms, and the transitions between these phases are tightly regulated [11]. However, the precise interactions and regulatory networks underlying these mechanisms remain poorly understood [12, 13]. One of emerging insights reveal that HS emanates from excessive dermal inflammation [14, 15]. Previous studies have shown a significant increase in pro‐inflammatory cytokine levels in HS tissue and activation of inflammasomes [16]. It is noteworthy that early post‐injury inflammation plays a pivotal role in shaping these outcomes [3, 17]. Hence, interventions directed at mitigating inflammatory responses at the site of injury may constitute efficacious measures for the prevention of HS. Several drugs, such as aspirin and paclitaxel, have been reported to be effective in suppressing scar formation in patients or animal models due to their anti‐inflammatory effects [18].

Our previous study found that an increased recruitment of macrophage lineage cells within scar tissue in the mouse scar model [19]. As integral elements in the body's restoration of tissue function post‐injury, macrophage lineage cells could rapidly respond to the damaged tissue microenvironment, ensuring optimal wound healing responses [20]. During this process, macrophages undergo significant metabolic reprogramming, which is closely linked to changes in mitochondrial dynamics [21, 22]. Mitochondrial fission, a process crucial for maintaining the normal morphology, distribution, and function of mitochondria, is essential for cellular homeostasis [23]. In macrophages, mitochondrial fission is dynamically regulated by inflammatory signals [24, 25], excessive mitochondrial fission in macrophages has been associated with a series of inflammatory cascade in response to challenges such as lipopolysaccharide (LPS) exposure [26]. Excessive mitochondrial fission can activate signaling pathways like NF‐κB and MAPK. Additionally, It has been reported that excessive mitochondrial fission in macrophages can augment aerobic glycolysis and reactive oxygen species (ROS) production, contributing to the “inflammatory storm” [27]. Recent years, dysregulated mitochondrial fission has been studied in various diseases, including neurodegeneration [28], cardiovascular diseases [29] cancer [30], and sepsis [27]. Nevertheless, the precise role of excessive mitochondrial fission in HS formation remains unknown.

Aurora kinase B (AURKB), traditionally recognized as a core regulator of mitotic processes such as chromosome segregation and cytokinesis [31], has recently been linked to non‐mitotic functions including oxidative stress responses and inflammatory signaling such as cGAS‐STING pathway [32].In this study, we first clarified that phosphorylation of DRP1 at Ser616 is a pivotal factor inducing excessive mitochondrial fission in macrophages within the skin wound inflammatory microenvironment. Furthermore, we identified that AURKB acts as an upstream regulator of DRP1, playing a critical role in modulating its phosphorylation level—a previously unrecognized regulatory mechanism bridging AURKB to macrophage mitochondrial dysfunction in wound inflammation. To translate these mechanistic insights into potential therapeutic strategies, we further performed high‐throughput screening of AURKB‐targeted small‐molecule drugs using machine learning approaches based on the ZINC20 database, and identified asiaticoside (AS) as a promising candidate.

**SCHEME 1:**
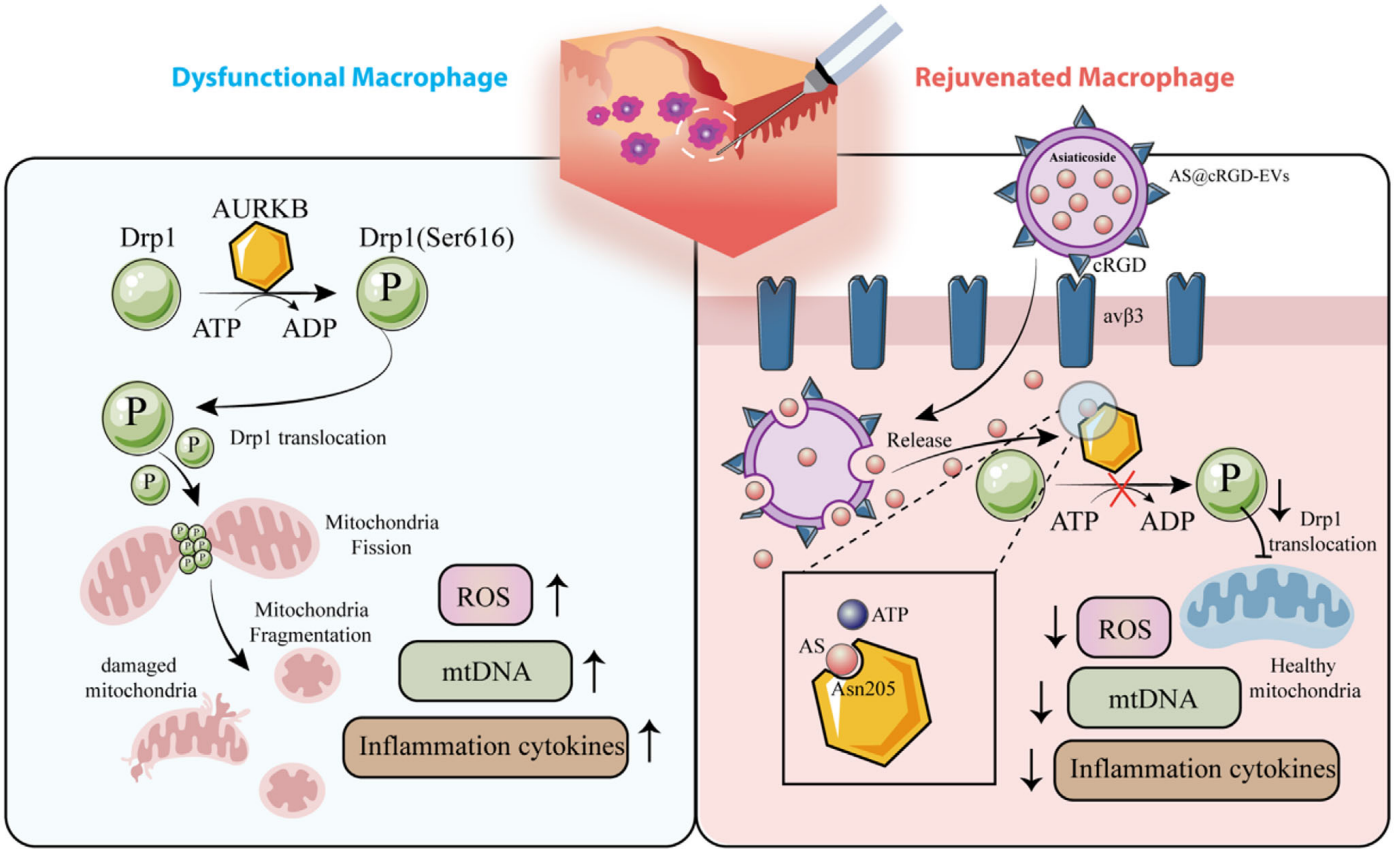
Targeted EVs deliver Asiaticoside to inhibit AURKB/DRP1‐mediated mitochondrial fission and attenuate hypertrophic scar formation.

AS, a triterpene saponin derived from natural herb *Centella asiatica* [33], has demonstrated significant therapeutic potential in various medical applications, such as promoting wound healing, inhibiting scar formation, and exerting anti‐inflammatory effects [34]. However, the clinical application of AS is significantly constrained by its high molecular weight (959.12 g mol^−1^), poor aqueous solubility, and low lipophilicity, which severely limit its transdermal absorption and result in low target‐site bioavailability [35]. Extracellular vesicles (EVs) are a class of lipid membrane vesicles approximately 50–150 nm in diameter, serving as important components of intercellular communication [36]. EVs exhibit superior biocompatibility with minimal immune activation and negligible cytotoxic effects. Their inherent phospholipid membrane architecture provides an optimal foundation for developing efficient in vivo drug delivery systems [37]. Engineered EVs can improve the tissue targeting capability following structural modifications, leading to enhanced treatment outcomes [38, 39, 40]. Therefore, in view of the application challenges of AS, we have developed an EV‐encapsulated AS targeted delivery system, which uses cRGD peptide‐modified EVs as the main carrier and can specifically enhance the uptake of the drug by macrophages in wound tissues.​ Overall, this delivery system can maximize the safe and effective targeted action of AS on macrophages, achieving precise anti‐inflammatory effects. Our hypothesis and workflow are shown below (Scheme 1).

## 2 Materials and Methods

### 2.1 Regents

Asiaticoside (AS, C_48_H_78_O_19_, molecular weight = 959.12, purity = 99.84%) (Catalog No: HY‐N0439), Mdivi‐1 (Catalog No: HY‐15886), and Barasertib (Catalog No: HY‐10127) were purchased from MedchemExpress (MCE, USA). Lipopolysaccharide (LPS) was purchased from Beyotime (Catalog No. S1732).

### 2.2 Isolation of Primary Scar Macrophages

Primary scar macrophages were isolated from mouse scar tissues using magnetic separation (MACS) Technology (Miltenyi) according to the manufacturer's instructions. Briefly, scar tissues from same group were pooled to form a single sample. After dissecting and removing meninges, tissues were dissociated using Multi Tissue Dissociation Kits 1 (130‐110‐201, Miltenyi). CD11b‐positive macrophages were magnetically labeled with CD11b MicroBeads, separated using a MACS Column (Miltenyi), and centrifuged at 400 g at 4°C for 5 min. The supernatant was discarded, and the isolated macrophages were retained for subsequent use.

### 2.3 Cell Culture

Mouse mononuclear macrophage cells Raw 264.7 (CSTR:19375.09.3101MOUSCSP5036) were generously provided by Prof. Yong Zuo (Department of Biochemistry and Molecular Cell Biology, Shanghai Jiao Tong University School of Medicine). For LPS stimulation, cells were treated with LPS (0.1 µg mL^−1^) following the protocol established by Li et al., [41]. Primary macrophages were isolated from mouse scar tissues via MACS. Raw 264.7 cells and primary macrophage were maintained in high‐glucose DMEM medium, supplemented with 10% fetal bovine serum (FBS) and 1% streptomycin/penicillin. All cells were cultured at 37°C in an incubator with humidified atmosphere of 5% CO_2_.

### 2.4 Mitochondrial Morphology Assessment

MitoTracker Red (C1049B, Beyotime) is a cell‐permeable X‐Rosamine derivative designed to selectively label active mitochondria within cells. After treating the cells with this dye at a concentration of 100 nM, we employed laser scanning confocal microscopy (LSCM) with a Zeiss980 microscope (ZEISS, Germany) to visualize mitochondrial morphology. Morphological analysis was performed using ImageJ software (National Institutes of Health, the USA).

### 2.5 Western Blot and Co‐Immunoprecipitation (Co‐IP)

Protein samples were isolated from cellular and tissue sources using RIPA lysis buffer (PC101, Epizyme) with subsequent quantification performed via BCA protein assay (ZJ102, Epizyme). Following denaturation, protein separation was achieved through SDS‐PAGE (7.5% or 12.5% gels) and electrotransferred onto PVDF membranes (IPVH00010, Millipore). Membranes were incubated with primary antibodies specific to: DRP1 (4E11B11, Cell Signaling, 1:1000), phospho‐DRP1 Ser616 (AF8470, Affinity, 1:1000), phospho‐DRP1 Ser637 (DF2980, Affinity, 1:1000), AURKB (3094T, Cell Signaling, 1:1000), GAPDH (60004‐1‐Ig, Proteintech, 1:50000), and β‐tubulin (10094‐1‐AP, Proteintech, 1:10000). Detection was accomplished using HRP‐conjugated secondary antibodies (A0208 and A0216, Beyotime, 1:1000). For co‐IP experiments, protein lysates were prepared with IP lysis buffer (P0013, Beyotime) and pre‐cleared by incubation with Protein A/G magnetic beads (HY‐K0202, MedChemExpress) at 4°C for 2 h. Primary antibody incubation was conducted overnight at 4°C, followed by immune complex isolation using magnetic beads and subsequent Western blot analysis. Rabbit IgG (30000‐0‐AP, Proteintech, 1 µg) served as the negative control in these experiments.

### 2.6 Transmission Electron Microscopy (TEM)

For TEM analysis, scars tissues from human and mouse were expeditiously fixed in a 2.5% glutaraldehyde solution at 4°C for a duration of 2 h. Subsequent to fixation, the samples were subjected to three rinses with a phosphoric acid rinse solution. Thereafter, the tissue specimens were immersed in a 1% osmic acid solution for 2 h. Following the fixation and staining procedures, the samples underwent a series of sequential steps, including dehydration, embedding, and solidification. Ultrathin sections with a thickness of 70 nm were generated using an ultramicrotome (EMUC7, Leica, Germany). These sections were further stained with a solution containing 2% uranyl acetate and lead citrate. Observations and image capture were conducted using a transmission electron microscope (TEM, ht7800) operating at 80 kV.

### 2.7 Inflammation Cytokine Arrays and Enzyme Linked Immunosorbent Assay (ELISA)

The Quantibody Mouse Interleukin Array (RayBiotech, Norcross, GA) was employed to quantitatively assess twenty murine cytokines in both Raw264.7 cell supernatants and wound tissue samples. Following manufacturer's protocols, cytokine levels were determined using specialized antibody pairs, with statistical significance set at *p* < 0.05. Data visualization was performed using R software (v4.3.1) to generate comprehensive heatmaps for pattern recognition. For targeted cytokine analysis, TGF‐β, IL‐6 and TNF‐α secretion was specifically evaluated by ELISA. Post‐treatment cell culture media were centrifuged (400 × *g*, 5 min, 4°C), with clarified supernatants analyzed using commercial ELISA kits (Mouse IL‐6: RK00008; Mouse TNF‐α: RK00027; ABclonal) to obtain quantitative measurements.

### 2.8 Measurement of ROS and Mitochondrial DNA (mtDNA)

ROS levels were determined utilizing 2',7'‐Dichlorodihydrofluorescein diacetate (DCFH‐DA) (Sigma, CAS No: 2044‐85‐1). DCFH‐DA is a fluorescent probe commonly used to measure intracellular ROS levels. To determine whether mtDNA is released into the cytosol upon LPS stimulation, mtDNA was detected by co‐expression of the mtDNA‐binding protein transcription factor A mitochondrial (TFAM, 22586‐1‐AP, 1:100, Proteintech), which was tagged with MitoTracker Red. ROS levels measurements were taken with a Flow cytometer, and results were calculated with FlowJo 10.4.1 software (Tree Star, Ashland, OR). and ImageJ software. The intensity of mtDNA within cells across all experimental groups was detected using a Zeiss880 fluorescent microscope.

### 2.9 Mitochondrial Membrane Potential Evaluation

To assess the mitochondrial membrane potential (MMP) in macrophages, cells were resuspended in PBS and analyzed using a FACScalibur cell analyzer (Becton Dickinson) equipped with a 488 nm argon laSer. Emission from the JC‐1 monomer was detected using a 530/30 nm band‐pass filter in FL‐1, while emission from the JC‐1 aggregate was detected using a 585/42 nm band‐pass filter in FL2. Data analysis was performed using Flowjo 10.4.1 software.

### 2.10 Transcriptome Analysis by Second‐Generation Sequencing

Transcriptome analysis was performed following Illumina's standard protocols using the TruSeq RNA Sample Preparation Kit with 1 µg total RNA input. The cDNA library preparation included end‐repair, phosphorylation, and A‐tailing steps prior to quantification with TBS380 and paired‐end sequencing (2×150 bp) on the NovaSeq 6000 platform. Read mapping and transcript assembly were conducted using StringTie's reference‐based approach (Pertea et al., Nat Biotechnol 2015, 33:290‐295). The study analyzed three matched pairs of HS and normal scar (NS) samples from RSM and sham groups. Mitochondrial fission‐related pathways were interrogated using GSEA (www.gsea‐msigdb.org), with gene importance evaluated by the MeanDecreaseGini algorithm (Calle & Urrea, 2011). All subsequent statistical analyses and data visualization were implemented in R (v4.3.1), maintaining rigorous quality control throughout the analytical pipeline. This comprehensive approach ensured robust detection of transcriptomic alterations associated with mitochondrial dynamics in scar formation.

### 2.11 Protein Expression and Purification

To obtain purified protein, characteristic plasmids with His tags (pCzn1 His prokaryotic expression vector) was constructed. Add 1 µL Plasmid to 100 µL‐competent bacteria, and incubate in 37°C overnight, making recombinant vector transform into Escherichia coli Arctic Express. The expression of recombinant bacterial fusion protein was induced by IPTG (isopropyl β‐D‐thiogalactoside). 12% SDS‐PAGE analysis and Coomassie Brilliant Blue staining were carried out to show bands. Utilizing a low‐pressure chromatography system, purified protein solution was collected and add it to a dialysis bag for further experiment. The gene sequence, synthesis was shown in Table S4, protein expression identification processes, and SDS‐PAGE analysis are in Supporting Information and Figure S2.

### 2.12 Microscale Thermophoresis (MST) Binding Affinity Assessment

To determine the binding affinity between AC and purified AURKB protein, we conducted an MST assay. Initially, a purified AURKB protein solution with a concentration of 10 µm was labeled with a dye at a concentration of 30 µm. Subsequently, 10 µL of gradient‐diluted AC (ranging from 3.05176E‐08 m to 0.0005 m) was mixed with 10 µL of the 100 nm labeled AURKB protein solution, allowing them to react for 5 min. The resulting mixture was loaded into a glass capillary and analyzed using the MST‐NT.115 equipment. Binding affinity analysis was performed using v2.3 NT software, and the dissociation constant (*K*d) was determined through curve fitting.

### 2.13 Ligand Discovery and Molecular Docking

To identify a purchasable lead compound suitable for intradermal injection, we conducted a target‐focused virtual screening against AURKB. A screening library was built from the ZINC20 “In‐Stock” subset, filtered for synthetic feasibility and structural diversity. A wide molecular weight range (250–1200 Da) was allowed to accommodate molecules suitable for local delivery. Affinity Prediction. Binding affinities were predicted using the pre‐trained MPNN‐CNN model in DeepPurpose (version 0.1.2). The model was fine‐tuned on our dataset with a target‐stratified 70/15/15 split, using a learning rate of 1e‐4, batch size 128, and weighted loss to handle class imbalance. Model performance was evaluated by AUROC (0.92) on a held‐out test set. Candidate Selection. Compounds were ranked by predicted Ki value. From the top‐ranked candidates (Ki < 100 nm). All computations used a fixed random seed (42). A schematic diagram illustrating the architecture and data flow of the MPNN‐CNN model is provided in Figure S5. For molecular docking analyses, the ligand and target protein structures were acquired and prepared. The construction of docking grid boxes for the protein structures was executed using PyMOL software (Schrodinger, the USA) and AutodockTools (The Scripps Research Institute, the USA). Subsequently, AutoDock Vina (The Scripps Research Institute, the USA) was employed to perform molecular docking assessments specifically for ligands. To gain insights into the 3D and 2D interaction forces, as well as to visualize these interactions, comprehensive analyses were conducted using PyMOL and Discovery Studio software. Molecular docking calculations were performed 9 times. The drug‐target binding affinity were represented as mean ± SD. If the binding energy is below ‐5 kcal mol^−1^, it suggests that the compound has a certain level of binding activity with the target (Gaillard, 2018).

### 2.14 Kinase Activity Assay

The LANCE Ultra Kinase Assay was utilized to evaluate kinase activity. This entailed the dilution of the kinase, ATP, inhibitors, and substrate in Kinase Buffer. The EU anti‐phosphotyrosine antibody was diluted to 8 nm in 1x LANCE detection buffer, creating a 4x detection mixture. The assay was conducted in optiplate‐384 wells using kinase inhibitors or kinase buffer, as well as the ulight‐crer tide/ATP mixture. After incubation and reaction termination, the detection mixture was introduced, followed by another incubation step. The resulting signal was measured in TR‐FRET mode using a nivo reader equipped with specific wavelengths. Data analysis and IC50 curve fitting were performed using Prism software.

### 2.15 Immunofluorescence (IF) Staining

To demonstrated the sub‐cell location and co‐localization of the target proteins, the cells with different treatments cultured in the confocal dishes were grown up to 70–80% confluency. After removed the medium and washed with PBS gently for 3 times, cells were permeabilized with PBST (0.5% Triton X‐100) for 30 min. After permeation, the dishes were soaked with PBS 3 times for 3 min each time, and goat Serum was added dropwise on the dishes bottom, and the room temperature was closed for 30 min. FlexAble CoraLite488 Antibody Labeling Kit for Rabbit IgG (KFA001, proteintech, China) and FlexAble CoraLite Plus 647 Antibody Labeling Kit for Rabbit IgG (KFA003, proteintech, China) were applied to label the primary antibody according instructions. Then Antibodies labeled with the FlexAble kit incubated with cells on the dishes bottom for overnight at 4°C, washed twice with PBS and stained dropwise with DAPI for 5 min. Laser scanning confocal microscopy (Zeiss LSM880, Germany) was used to image the cells.

### 2.16 Preparation of AS@cRGD‐EVs

Natural EVs were isolated from HEK293T cell supernatants through differential centrifugation (500×*g*/5 min → 2000×*g*/30 min → 10 000×*g*/60 min → 120 000×*g*/70 min, all at 4°C), followed by 0.22 µm filtration and PBS resuspension. For targeted delivery, EVs were functionalized with cRGD‐liposomes via membrane extrusion (200 nm polycarbonate), then loaded with AS (500 mg L^−1^) using electroporation (4 mm cuvette). After overnight incubation (4°C) and ultracentrifugation (200 000×*g*/2 h) to remove unencapsulated AS, the resulting AS@cRGD‐EVs were characterized by NTA and TEM. The AS@cRGD‐EVs suspension after incubation was subjected to ultracentrifugation (100 000×*g*, 4°C, 2 h) to completely separate the supernatant (free AS) from the pellet. The supernatant was collected, and the concentration of free AS was determined by ultraviolet spectrophotometry at 205 nm, with the mass of free AS (*m*free) calculated based on the measured concentration and supernatant volume. The encapsulation efficiency was calculated using the formula:

$$\left(\mathrm{EE},\%\right)=1-\frac{m\mathrm{total}\;}{m\mathrm{free}}\;*100\%$$

For drug loading calculation, the initial mass of blank EVs (mexo) was quantified by BCA protein assay. The drug loading was derived from the obtained EE value using the formula:

$$\left(\mathrm{DL},\%\right)=\frac{\mathrm{EE}\times m\mathrm{total}}{\mathrm{mexo}+\mathrm{EE}\;\times \;m\mathrm{total}}\;*100\%$$

The in vitro release of AS from cRGD‐EVs was assessed by gentle stirring in PBS (pH 7.4) at 37°C. Samples were collected at 1, 2, 4, 8, and 24 h, and AS release was quantified as previously described.

### 2.17 Hemocompatibility

Incubate AS@cRGD‐EVs with diluted human whole blood red blood cell suspension at 37°C for a certain period of time for 4 h, centrifuge, and take the supernatant. Measure the absorbance (characteristic absorption peak of hemoglobin) at 540 nm using an enzyme‐linked immunosorbent assay reader.

### 2.18 Stability Evaluation

For stability evaluation, AS@cRGD‐EVs formulations were subjected to different pH conditions (4.0, 7.0, 10.0) at 37°C and various temperatures (‐80°C, 4°C, 25°C, 37°C) in PBS. Size and PDI were measured at 72 h points using dynamic light scattering (DLS) to assess the physicochemical stability of the exosome formulation under these conditions.

### 2.19 Studies in Animals

To evaluate the in vivo therapeutic efficacy of AS@cRGD‐EVs, a hypertrophic scar model named RSM was established on the dorsal skin of mouse as previous study [19]. All animal experiments were conducted in accordance with protocols approved by the Animal Ethics Committee of Shanghai Ninth People's Hospital, Shanghai Jiao Tong University School of Medicine (SH9H‐2020‐A314‐1, March 14, 2020). Briefly, male C57BL/6 mice were anesthetized, and a 1 cm longitudinal full‐thickness skin incision was made on the dorsum, followed by suturing. Mice in the sham group healed naturally after suture removal, while those in the recurrent scar model (RSM) group were subjected to a skin‐reversing and tension‐applying device post‐suture removal to induce hypertrophic scarring. For therapeutic intervention, AS@cRGD‐EVs were prepared by incubating cRGD‐modified EVs (1 mg mL^−1^) with AS (500 µg mL^−1^), followed by purification to remove unencapsulated drug, yielding a drug loading efficiency of 16.9%. To achieve a therapeutically relevant dose within the volume constraints of intradermal injection, the purified AS@cRGD‐EVs were concentrated 10‐fold to a final exosomal protein concentration of 10 mg mL^−1^. Mice in the treatment group received daily intradermal injections of this formulation—administered as two 20 µL injections per lesion for a total of 40 µL daily, delivering approximately 33.8 µg of AS per lesion per day.

### 2.20 Histology

Routine hematoxylin‐eosin (HE) staining to evaluate the cross‐sectional scar area and the Masson staining to assess the density of collagen fibers. Samples were performed on 5 µm thick paraffin‐embedded sections. The differences among different treatments were assessed using pathologic section scanner. The results were also analyzed via ImageJ software. The scar elevation index (SEI) was calculated according to equation:

$$\mathrm{SEI}=H/H_{0}$$

where *H*_0_ represents the distance from the stratum corneum of the surrounding normal skin to the subcutaneous tissue, and *H* represents the distance between the thickest stratum corneum in the hypertrophic scar and the subcutaneous tissue.

### 2.21 Statistical Analysis

Statistical analysis was performed using specialized software (GraphPad Prism 9.0). Quantitative data are expressed as either arithmetic mean ± SD or median with interquartile ranges, depending on distribution characteristics and variance homogeneity. Intergroup comparisons were conducted using parametric (Student's t‐test for pairwise comparisons; one‐way ANOVA for multiple groups) or nonparametric tests as appropriate, with post‐hoc analyses when indicated. Statistical significance was defined at the conventional threshold of *p* < 0.05.

## 3 Results

### 3.1 Excessive Mitochondrial Fission and Inflammatory Response Were Found in HS‐Macrophages

**FIGURE 1:**
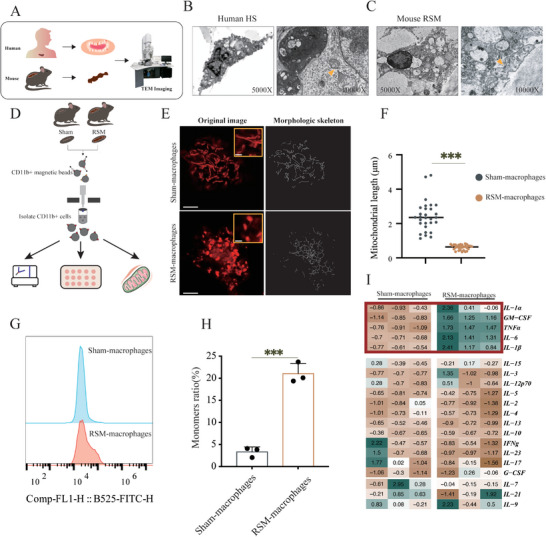
Excessive mitochondrial fission and inflammatory response were found in HS‐macrophages. (A) Schematic of hypertrophic scar (HS) collection from HS patients and mouse RSM for TEM analysis. (B,C) TEM image of a macrophage from human HS and mouse HS, shows mitochondria undergoing fission in HS macrophages (yellow arrows) (D) Schematic for isolating sham‐macrophages and RSM‐macrophages via MACS. (E) Mito‐Tracker Red staining images and morphology analysis of sham‐macrophages and RSM‐macrophages, scale bar = 5 µm, with yellow insets showing magnified mitochondria within macrophages, scale bar = 1 µm. (F) Quantitative analysis of mitochondrial lengths in sham‐macrophages and RSM‐macrophages. (G) Flow cytometry for JC‐1 monomers in sham‐macrophages and RSM‐macrophages. (H) Quantitative analysis of monomers ratio according to flow cytometry results. (I) Heatmap of inflammatory cytokines expression in sham‐macrophages and RSM‐macrophages. (*n* = 3, *p < 0.05, **p < 0.01, ***p < 0.001, NS = not significant).

Building upon our prior finding of increased macrophage recruitment in the RSM model [19], we further observed that RSM wound tissues contained significantly more resident macrophages (*p* < 0.001, Figure S4A,B) and exhibited elevated levels of pro‐fibrotic factors, including TGF‐β, compared to the Sham group (*p* < 0.001, Figure S4C). These results prompted us to investigate macrophage ultrastructure for mechanistic insights into scar formation. TEM analysis of scar tissues from both human HS patients and RSM mice revealed extensive mitochondrial fragmentation within macrophages (Figure 1A–C). Consistently, primary macrophages isolated from RSM scar tissue via MACS possessed significantly shorter mitochondria than those from Sham controls (*p* < 0.001, Figure 1D–F). Morphologically, mitochondria in RSM‐macrophages shifted from the tubular form (2–4 µm) seen in Sham cells to a predominantly granular morphology (0.2–1 µm). We next assessed mitochondrial function and inflammatory status. Flow cytometry indicated a loss of mitochondrial membrane potential in RSM‐macrophages, evidenced by an increase in JC‐1 monomers (*p* < 0.001, Figure 1G,H). Furthermore, a cytokine array showed that RSM‐macrophages secreted higher levels of key pro‐inflammatory cytokines, including IL‐1α, GM‐CSF, TNF‐α, IL‐6, and IL‐1β (Figure 1I).

### 3.2 The Phosphorylation Level of DRP1 at Ser616 Was Increased in LPS‐Induced Macrophages

**FIGURE 2:**
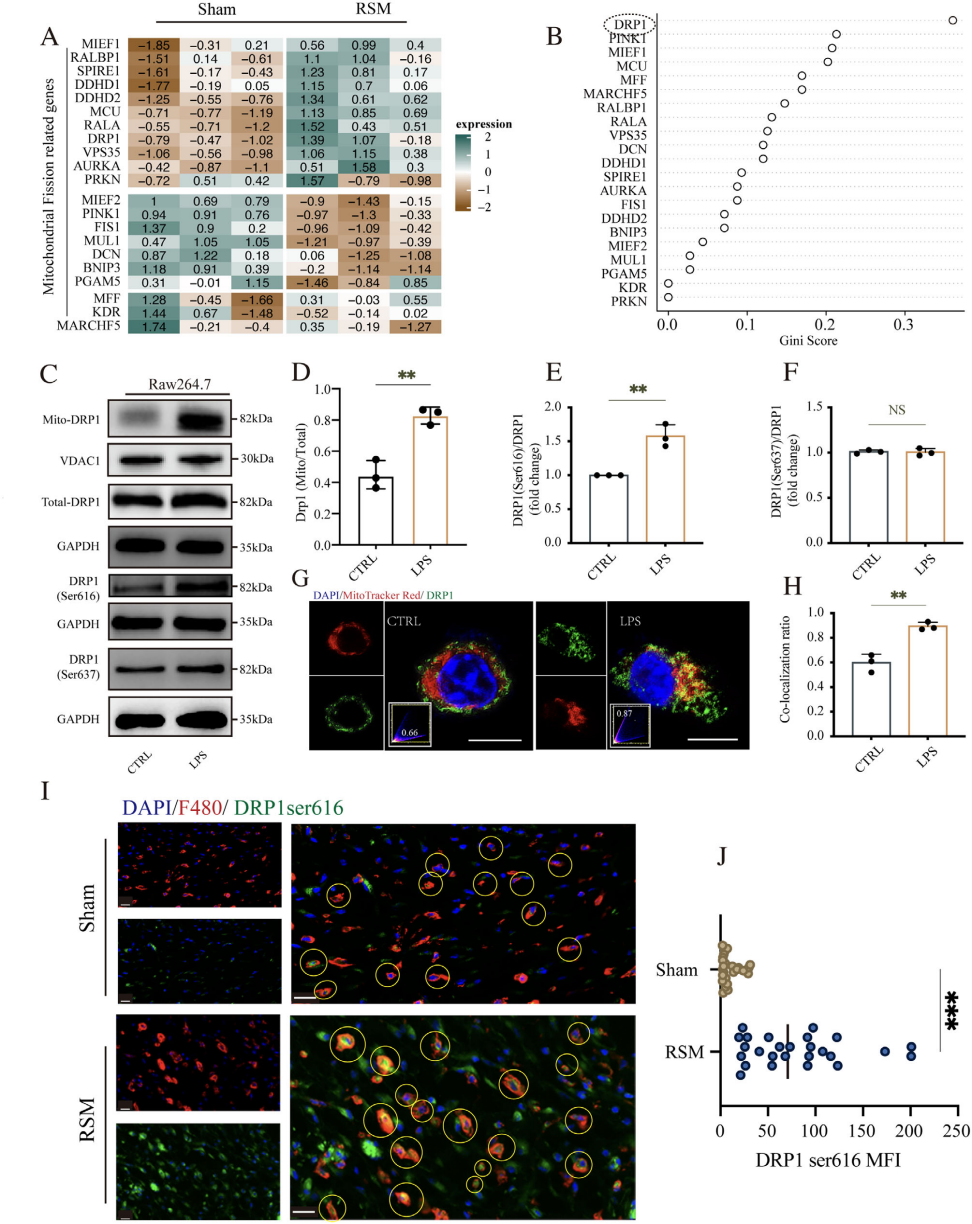
The phosphorylation level of DRP1 at Ser616 was increased in LPS‐induced macrophages. (A) Heatmap illustrating the expression levels of 21 mitochondrial fission‐related genes in scars from sham groups and RSM groups. (B) Random forest algorithm output ranking the importance Gini score of identified genes in mitochondrial fission. (C) Western blot analysis of DRP1 and its phosphorylation at Ser637 and Ser616. (D) Quantification of DRP1 relative expression level (fold change) in mitochondria and total protein. (E,F) Quantification of relative ratio of DRP1(Ser616)/DRP1 and DRP1(Ser637)/DRP1. (G,H) Confocal microscopy images showing the localization of DRP1ser616 (green) in macrophages, scale bar = 10 µm, small box indicating the co‐localization ratio with Pearson correlation coefficient. (I,J) Representative images and quantification of DRP1(Ser616) expression of macrophages from wound tissue in sham groups and RSM groups, scale bar = 200 µm. (*n* = 3, *p < 0.05, **p < 0.01, ***p < 0.001, NS = not significant).

The above results indicated that the progression of HS is associated with excessive mitochondrial fission. To elucidate the underlying molecular mechanisms, NS from sham groups and HS tissues from RSM groups were collected for second‐generation sequencing. As shown in Figure 2A, 21 differentially expressed genes (DEGs) related to mitochondrial fission were identified. Among these, the gene encoding dynamin‐related protein 1 (DRP1) was highlighted as a critical regulator, showing the highest Gini score in the Random Forest analysis (Figure 2B). Since DRP1‐mediated mitochondrial fission primarily occurs through its translocation to mitochondria [42], we first examined DRP1 levels in mitochondrial versus total protein of macrophages. We found that the ratio of mitochondrial DRP1 to total DRP1 was significantly increased following LPS stimulation (*p* < 0.01, Figure 2C,D). Additionally, immunofluorescence analysis confirmed a higher co‑localization of DRP1 with mitochondria in the LPS group compared to controls (*p* < 0.01, Figure 2G,H). Together, these results indicate that LPS induces significant translocation of DRP1 from the cytoplasm to mitochondria, thereby amplifying the mitochondrial fission process. Given that DRP1 translocation is regulated by phosphorylation, we focused on two key phospho‑sites, Ser616 and Ser637. Western blot analysis showed that LPS treatment significantly increased the DRP1(Ser616)/total DRP1 ratio (*p* < 0.01, Figure 2C,E), while the phosphorylation level at Ser637 remained unchanged (*p* > 0.05, Figure 2C,F). In line with this, immunofluorescence staining revealed stronger DRP1(Ser616) signals in macrophages from wound tissues of the RSM group compared to the sham group (*p* < 0.001, Figure 2I–J).

### 3.3 Inhibiting Mitochondrial Fission Modulated the Inflammatory Response in LPS‐Induced Macrophages

**FIGURE 3:**
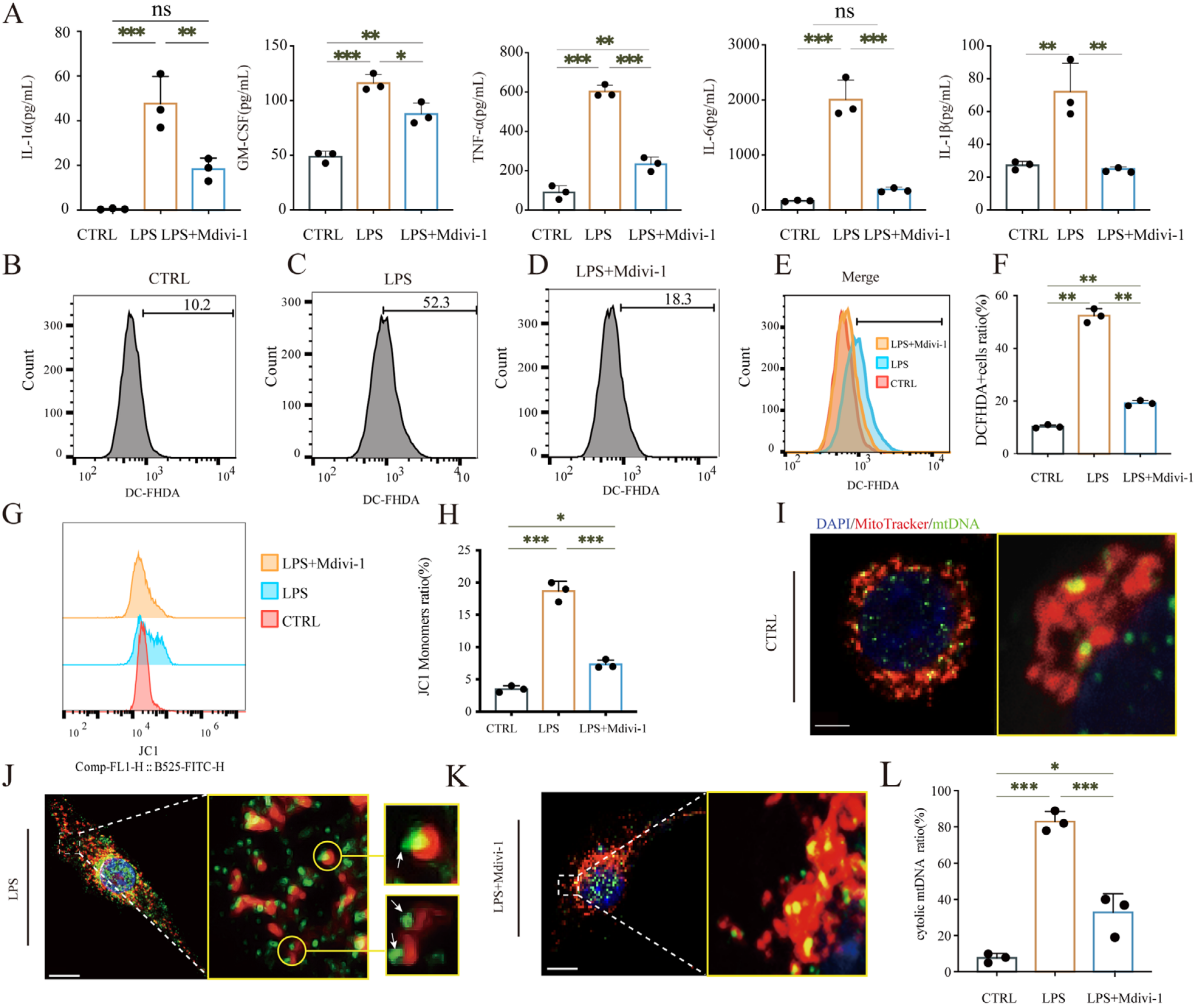
Inhibiting mitochondrial fission modulated the inflammatory response in LPS‐Induced macrophages. (A) Bar charts showing the quantification of cytokines. (B–D) Representative flow cytometry analysis showing ROS levels as indicated by DCFH‐DA fluorescence in control, LPS, and LPS + Mdivi‐1 treated macrophages. (E,F) Overlay histograms from flow cytometry demonstrate DCFH‐DA fluorescence across treatments and a bar chart of DCFH‐DA mean fluorescence. (G,H) Half offset histograms of flow cytometry for JC‐1 monomers and quantitative statistics of monomers rate in macrophages with different treatments. (I,K) Representative fluorescent images of macrophages stained for mitochondria (MitoTracker, red), and mitochondrial DNA (mtDNA, green), scale bar = 5 µm. The enlarged illustration shows the positional relationship between mtDNA and mitochondria in macrophages under different treatments, scale bar = 1 µm. (L) Bar chart summarizing the ratio of cytoplasmic to total mtDNA. (*n* = 3, *p < 0.05, **p < 0.01, ***p < 0.001, NS = not significant).

To clarify whether the inhibition of mitochondrial fission could improve the stability of mitochondria and regulate the inflammatory response during wound healing, LPS with or without Mdivi‐1 was used to treat macrophages Raw264.7 in vitro. As shown in Figure 3A, LPS significantly increased the level of inflammatory cytokines including IL‐1α (*p <* 0.001), GM‐CSF (*p <* 0.001), TNF‐α (*p <* 0.001), IL‐6 (*p <* 0.001), and IL‐1β (*p <* 0.01), while this effect was substantially reversed by Mdivi‐1. According to the result of flow cytometry analysis with DCFH‐DA fluorescent staining, the count of DCFH‐DA positive cells was higher in the LPS group than that in the LPS+Mdivi‐1 group (*p <* 0.01, Figure 3B–F). This indicated that ROS production was markedly elevated after LPS stimulation, while Mdivi‐1 partly reversed this effect. More JC‐1 became monomeric and fluoresced green in the LPS‐stimulated group compared to that in the control group, (*p <* 0.01, Figure 3G,H), while Mdivi‐1 prevented this decrease of MMP (*p <* 0.01, Figure 3G,H). Additionally, LPS stimulated more mtDNA efflux, from mitochondria to cytoplasm (*p <* 0.001, Figure 3I,J,L), and Mdivi‐1 significantly inhibited mtDNA efflux at the presence of LPS (*p <* 0.001, Figure 3K,L).

### 3.4 Aurora Kinase B (AURKB) Regulated the DRP1 Phosphorylation and Mitochondrial Fission in Macrophages

**FIGURE 4:**
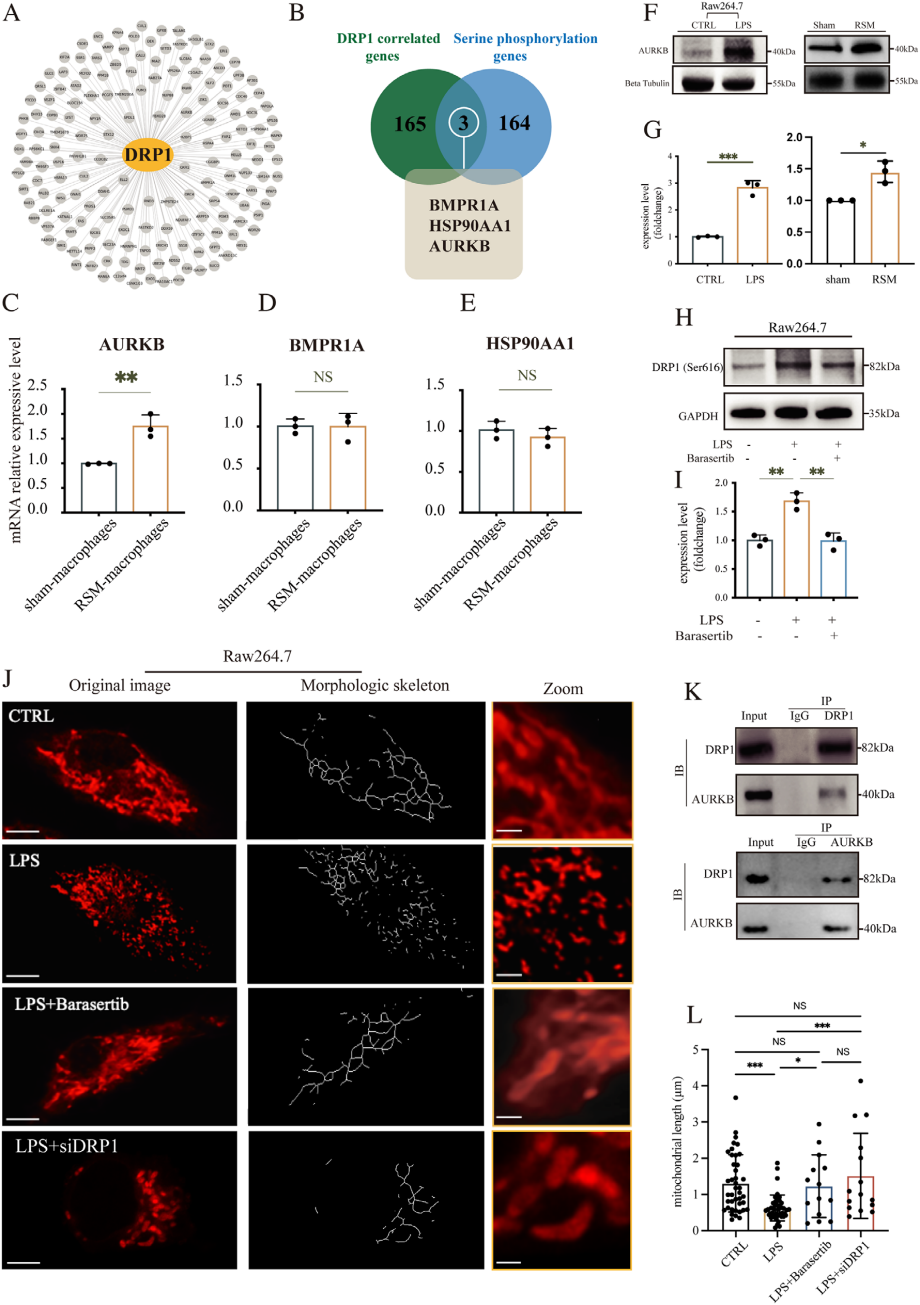
Aurora kinase B (AURKB) regulated the DRP1 phosphorylation and mitochondrial fission in macrophages. (A) Network diagram illustrating the gene's connections to DRP1. (B) Venn diagram showing the relationship between DRP1‐correlated genes and Serine phosphorylation genes. (C–E) Bar graphs depicting mRNA levels of AURKB, BMPR1A, and HSP90AA1. (F,G) Western blot results and analysis for AURKB in macrophages and scar tissues from mice. (H,I) Western blot showing phosphorylation of DRP1 at Serine 616 under LPS treatment and its modulation in the presence of the Aurora B inhibitor Barasertib. (J) MitoTracker Red staining image and morphology analysis of macrophages with different treatments, scale bar = 5 µm, with insets showing magnified mitochondria within macrophages, scale bar = 1 µm. (K) Co‐Immunoprecipitation (CO‐IP) between AURKB and DRP1 in macrophages. (L) Quantitative analysis of mitochondrial lengths in macrophages. (*n* = 3, *p < 0.05, **p < 0.01, ***p < 0.001, NS = not significant).

**FIGURE 5:**
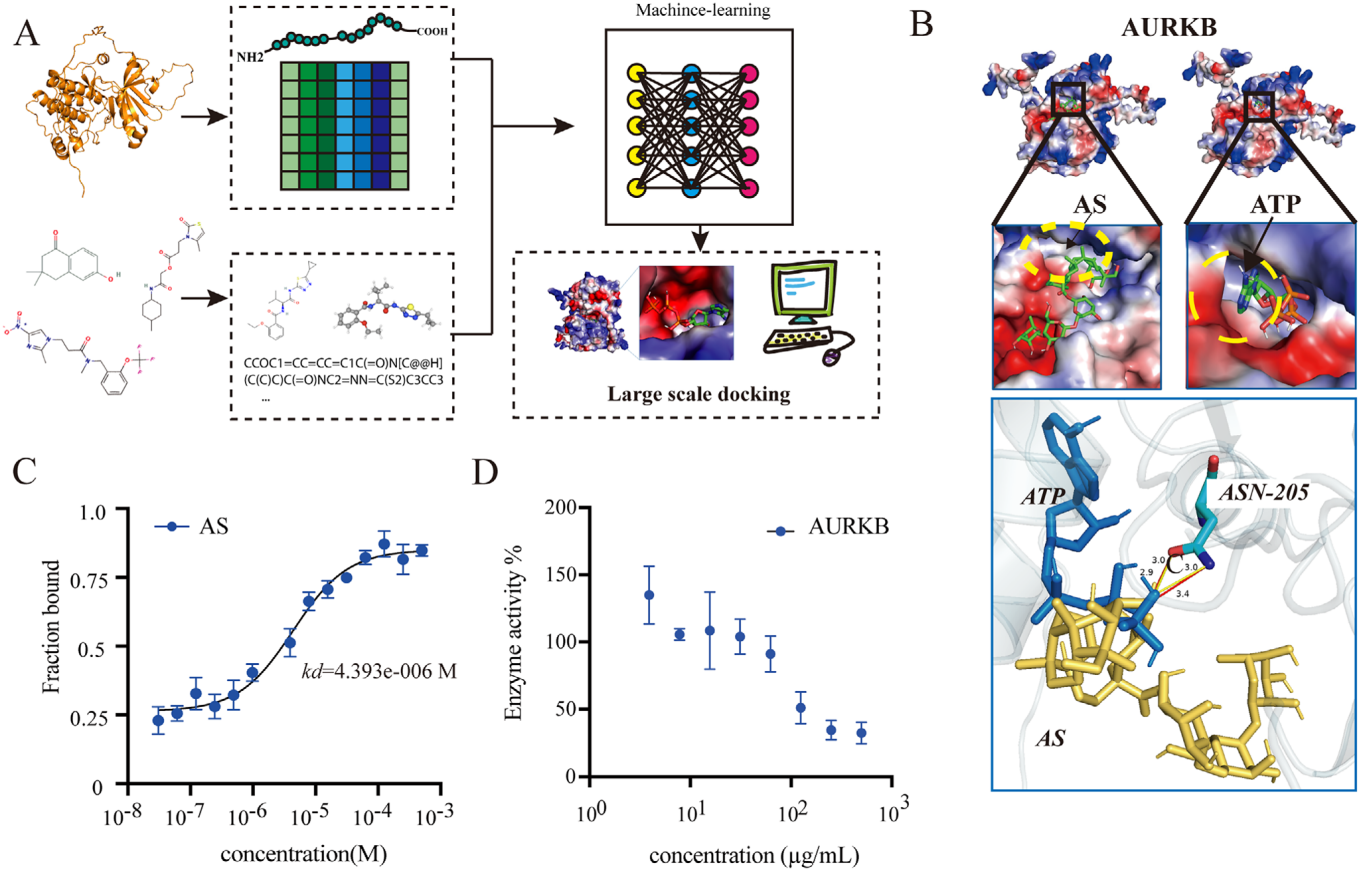
AS was identified as a potential modulator of AURKB. (A) Schematic representation of the machine learning process for identifying the potential ligand of AURKB. (B) Detailed view of AS and ATP molecular docking with AURKB, and insets display the molecular interactions within the binding site and the overlapping binding sites of AS and ATP on AURKB. (C) MST analysis of the binding affinity between AC and AURKB protein. (D) In vitro kinase activity inhibition assay of AS acting on AURKB.

To elucidate potential upstream regulators of DRP1(Ser616) phosphorylation, correlation gene‐set enrichment analysis was conducted. The network diagram illustrated genes that connected to DRP1 (Figure 4A). We then screened genes exhibiting high correlation with DRP1(correlation score>0.99) and conducted an intersect analysis with genes encoding Serine phosphorylation sites kinases. Finally, 3 genes including BMPR1A, HSP90AA1, and AURKB were identified as the potential upstream of DRP1(Ser616) (Figure 4B). Verification was conducted in primary mouse macrophages derived from NS in sham groups and HS in RSM groups. The results of RT‐qPCR revealed that the expression level of AURKB in the RSM‐macrophages was significantly increased (*p <* 0.01, Figure 4C), while no significant difference in the expression level of BMPR1A or HSP90AA1 was found between the sham‐macrophages and RSM‐macrophages(*p >* 0.05, Figure 4D,E). Western blot analysis revealed a significantly higher expression of AURKB in the LPS group compared to the control group (*p* < 0.001, Figure 4F,G). Consistently, in vivo studies demonstrated elevated AURKB expression in the RSM group relative to the sham group (*p* < 0.05, Figure 4F,G). As shown in Figure 5H,I, LPS significantly increased the expression level of DRP1(Ser616) (*p <* 0.01), while this effect was substantially reversed by a selective AURKB inhibitor Barasertib (*p <* 0.01). The interaction between AURKB and DRP1 was further confirmed by a Co‐IP assay (Figure 4K). Additionally, immunofluorescence results demonstrated their co‐localization in cells (Figure S4H). Furthermore, the length of mitochondria in the LPS group was significantly shorter than that in the CTRL group (*p* < 0.001, Figure 4J,L). While Barasertib significantly reversed this change (*p* < 0.05, Figure 4J,L), and knockdown of DRP1 yielded a comparable rescuing effect (*p* < 0.001, Figure 4J,L). The mitochondrial morphology in the LPS+siDRP1 group was not significantly different from that in the CTRL and LPS+Barasertib group, but was significantly different from the LPS group. These results collectively suggest that LPS promotes mitochondrial fission predominantly through AURKB‐DRP1 axis.

### 3.5 AS Was Identified as a Potential Modulator of AURKB

Based on machine learning‐assisted virtual screening of the ZINC20 database, we identified small molecules with predicted AURKB binding affinities (Ki) below 100 nm (Table S1) for subsequent experimental validation (Figure 5A). CCK‐8 assay showed that AS has a high cell compatibility without cytotoxic effects at concentrations up to 1 mm (Figure S1). To investigate the interaction between AS and AURKB, molecular docking simulations were conducted. Repeated molecular docking simulations for 9 times showed that binding affinity between AS and AURKB protein was ‐7.71 ± 0.44 kcal mol^−1^ (Table S2), and the average predicted binding value is 2.20 µm, indicating that AS can stably bind to the active site of AURKB. At the same time, the molecular docking between AURKB protein and ATP was performed, and the results showed that binding affinity between AURKB and ATP was ‐6.57 ± 0.25 kcal mol^−1^ (Table S3). Further comparison of the binding sites of AS and ATP with protein AURKB, the results revealed that AS and ATP share one binding site: ASN‐205 (Figure 5B). In vitro MST assay revealed that the dissociation constant (*K*_d_) value between AURKB and AS was 4.393 µm (Figure 5C), confirming a strong binding affinity between AS and AURKB. In vitro kinase activity testing also found that AS could inhibit AURKB kinase activity in a concentration‐dependent manner (Figure 5D).

### 3.6 Preparation and Characterization of Engineered AS@cRGD‐EVs

**FIGURE 6:**
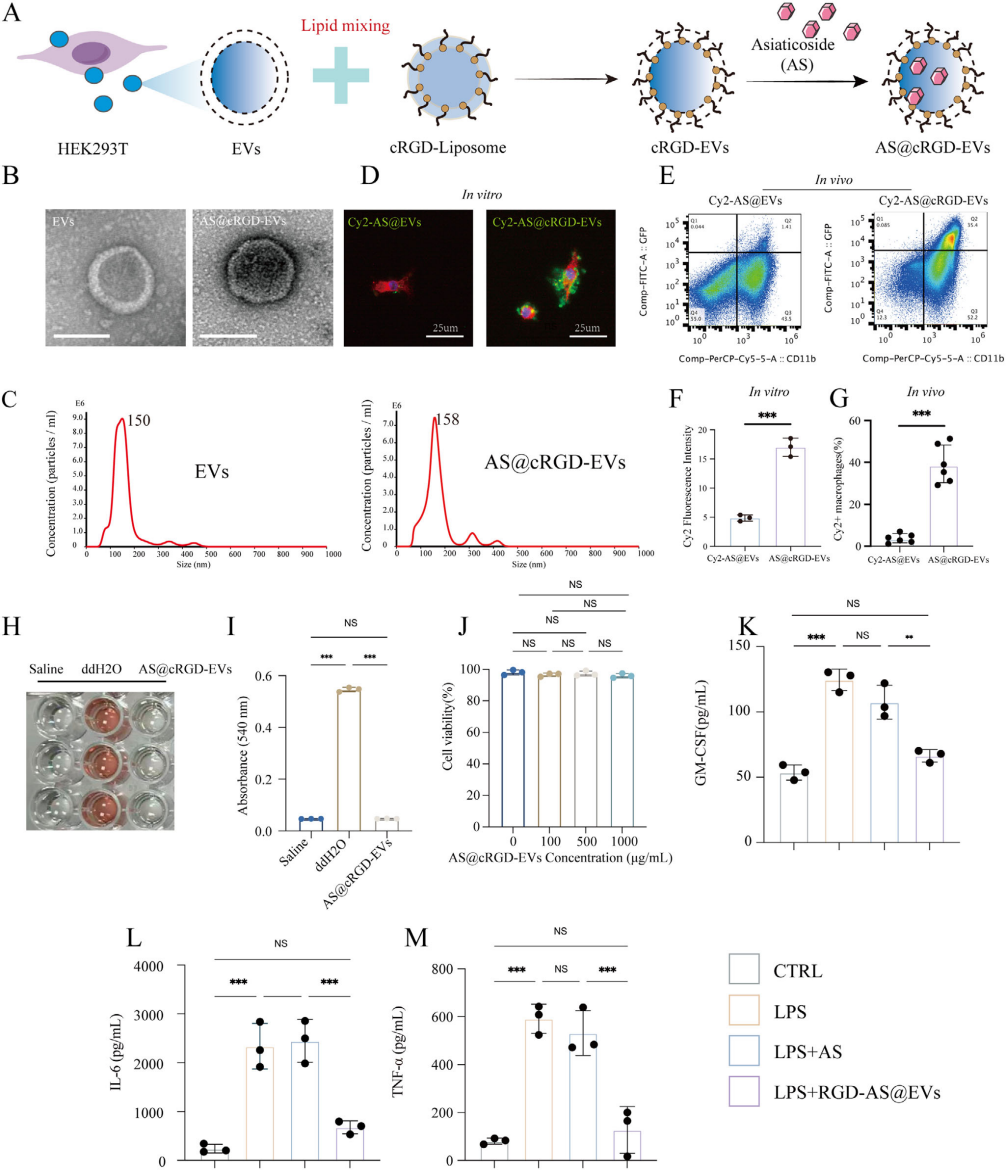
Preparation and characterization of engineered AS@cRGD‐EVs. (A) Schematic illustration of the preparation of engineered AS@cRGD‐EVs. (B) TEM images of extracellular vesicles (EVs) and engineered AS@cRGD‐EVs. Scale bars, 100 µm. (C) NTA measured the particle size of EVs and AS@cRGD‐EVs. (D, F) The immunofluorescence image of Cy2‐labeled AS being phagocytosed in macrophages, *n* = 3. Scale bars = 25 µm. (E, G) The flow cytometry analysis of Cy2‐labeled AS being phagocytosed in vivo, *n* = 6. (H–J) Hemolysis assays, *n* = 3 (H) and biocompatibility studies of AS@cRGD‐EVs, *n* = 3 (J). (K–M) Bar graphs displaying the concentrations of GM‐CSF, IL‐6, and TNF‐α via Elisa array, n = 3. (*p < 0.05, **p < 0.01, ***p < 0.001, NS = not significant).

AS is a natural active ingredient extracted from *Centella asiatica*. A large number of previous studies have shown that it has various pharmacological effects such as promoting wound healing, anti‐inflammation, anti‐oxidation [43]. However, its clinical application is often limited by its own physicochemical properties and delivery efficiency [35]. EVs, as natural nanoscale vesicle carriers [44], have the potential to solve the delivery problem of AS. We obtained HEK293T cells‐derived EVs by ultracentrifugation. In order to generate engineered EVs targeting macrophages in wounds tissue, cyclic RGD pentapeptide (Arg‐Gly‐Asp‐Phe‐Val) was selected to be engineered onto the surface of EVs, and AS was loaded into EVs through electroporation technology (Figure 6A), cRGD pentapeptide (Arg‐Gly‐Asp‐Phe‐Val) is a targeting moiety has been widely utilized to enhance the targeting and delivery efficiency to macrophages [45]. Meanwhile, single‐cell RNA sequencing (scRNA‐seq) data revealed the expression levels of integrin αv (ITGAV) (*p* < 0.001, Figure S4D) and β3 (ITGB3) (*p* < 0.05, Figure S4D) subunits in macrophages from the sham and RSM groups.

EVs exhibited a typical spherical vesicle morphology under TEM, AS@cRGD‐EVs also showed a spherical shape, and their surface was slightly rougher than that of un‐engineered EVs (Figure 6B), which might be related to the modification of RGD peptides at a relatively high density. NTA analysis showed that the average particle sizes of EVs and AS@cRGD‐EVs were 150 and 158 nm, respectively (Figure 6C). The EE% and DL% of AS@cRGD‐EVs plateaued at their maximum values (88% and 16.9%, respectively) at an AS concentration of 500 mg L^−1^ (Figure S4J). Therefore, this concentration was selected as the optimal loading condition to maximize exosome loading capacity while avoiding drug waste. AS@cRGD‐EVs exhibited stable z‐average particle size and PDI across pH 4–10, showing good pH tolerance (Figure 4SK). It also maintained consistent physicochemical properties from ‐80°C to 37°C (Figure S4K), confirming robust stability for storage and in vivo use. The release data of AS from EVs followed first‐order kinetics, characterized by an initial rapid release (22.65%) followed by a sustained phase reaching a plateau at 45.91% within 24 h, with a release constant (*K*) of 0.5173 h^−^^1^ (Figure S4L).

To evaluate the delivery efficiency of AS in vitro and in vivo, we labeled AS with the Cy2 fluorochrome. After 24 h of co‐culture with Raw264.7 macrophages, cells treated with AS@cRGD‐EVs displayed significantly stronger Cy2 fluorescence intensity compared to those treated with AS@EVs (*p* < 0.001, Figure 6D,F). To further assess in vivo targeting, we analyzed the proportion of Cy2‑positive macrophages in wound tissues from Sham and RSM groups by flow cytometry. Consistently, the percentage of Cy2^+^ macrophages was significantly higher in the AS@cRGD‐EVs group than in the AS@EVs group (*P* < 0.001, Figure 6E,G), indicating that the cRGD modification substantially enhanced AS delivery efficiency both in vitro and in vivo. Regarding safety test, we conducted hemolysis and biocompatibility assays. The results showed that AS@cRGD‐EVs did not induce hemolysis, with no significant difference compared to the normal saline group (Figure 6H,I). Furthermore, AS@cRGD‐EVs, at concentrations ranging from 0 to 1000 µg mL^−1^, exhibited no cytotoxicity (Figure 6J). Furthermore, we evaluated the inflammatory level in the cell supernatant. The results showed that free AS has no significant inhibitory effect on the release of GM—CSF, IL‐6, and TNF‐α (Figure 6K–M). And AS@cRGD ‐EVs exerted a remarkable inhibitory effect on the secretion of LPS‐induced GM—CSF (*p <* 0.01, Figure 6K), IL–6 (*p <* 0.001, Figure 6L), and TNF—α (*p <* 0.001, Figure 6M). These results suggest that the RGD‐targeted modification combined with the exosome delivery system can significantly enhance the anti‐inflammatory efficiency of AS.

### 3.7 Engineered AS@cRGD‐EVs Could Inhibit AURKB‐DRP1(Ser616) Mediated Mitochondrial Fission In Vitro

**FIGURE 7:**
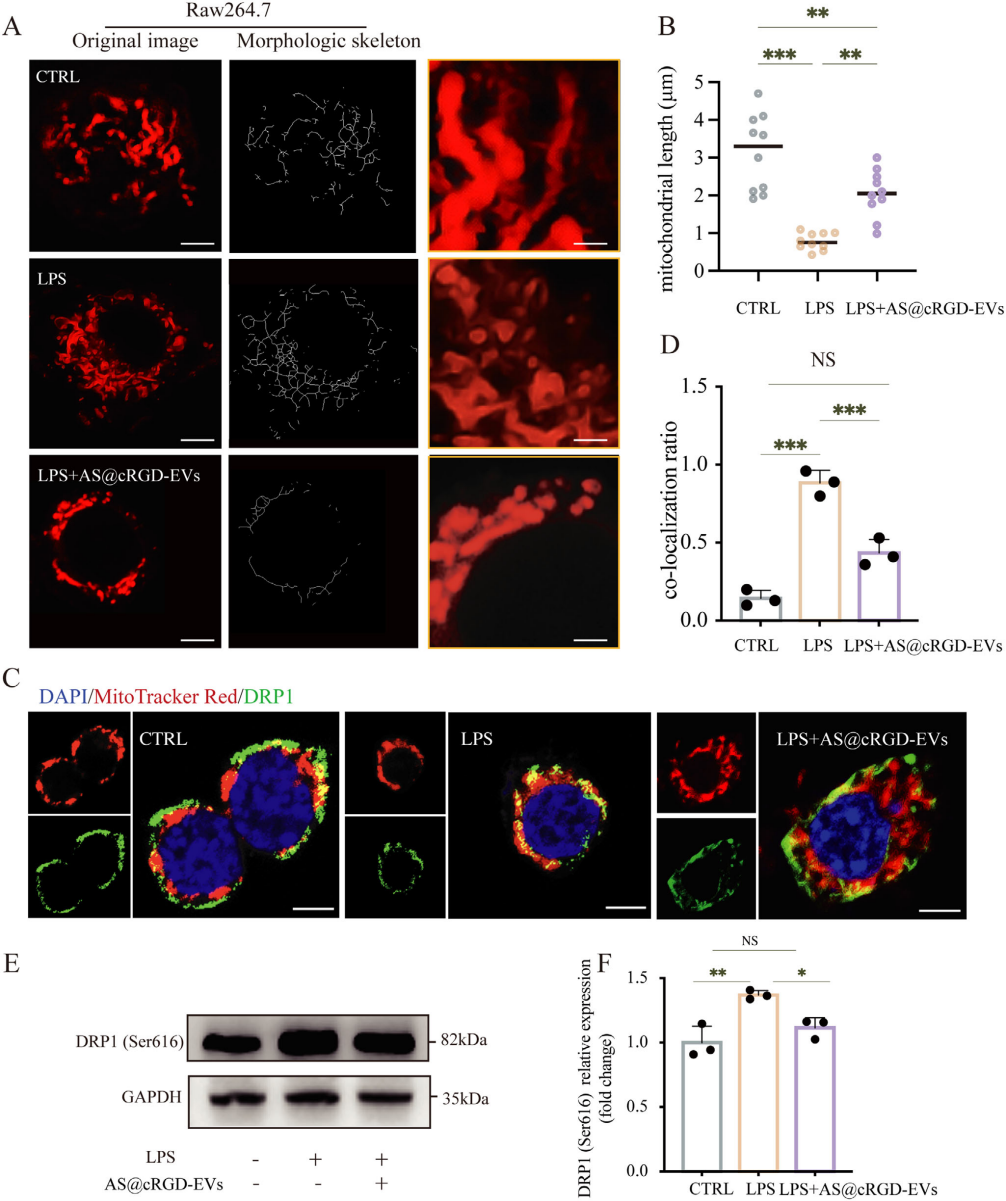
Engineered AS@cRGD‐EVs could inhibit AURKB‐DRP1(Ser616) mediated mitochondrial fission in vitro. (A) Fluorescence microscopy images and morphology analysis of mitochondria, scale bar = 5 µm, with insets providing a higher magnification view, scale bar = 1 µm. (B) Quantitative analysis of mitochondrial length across different treatment conditions. (C) Immunofluorescence staining for DRP1 (green) in cells, indicating the distribution of DRP1 in control, LPS, and LPS+ AS@cRGD‐EVs conditions, with the nucleus stained in blue. Insets show a higher magnification of DRP1 distribution, scale bar = 5 µm. (D) The co‐localization ratio of DRP1 with mitochondria. (E,F) Western blot images and semi‐quantitative analysis of DRP1(ser616) under different conditions. (*n* = 3, *p < 0.05, **p < 0.01, ***p < 0.001, NS = not significant).

To investigate whether AS@cRGD‐EVs can inhibit mitochondrial fission in macrophages, we randomly divided macrophages into three groups: control group, LPS‐stimulated group, and LPS‐stimulated + AS@cRGD‐EVs treatment group. After 24 h of treatment, MitoTracker Red staining was used to observe and analyze the mitochondrial morphology of macrophages in each group. The results showed that AS@cRGD‐EVs effectively reversed the excessive mitochondrial fission induced by LPS stimulation and maintained mitochondrial length to a certain extent (*p <* 0.01, Figure 7A,B). Furthermore, IF assay revealed that AS@cRGD‐EVs treatment significantly reduced the translocation ratio of DrDp1 to mitochondria after LPS stimulation (*p <* 0.001, Figure 7C,D). Meanwhile, Western blot results demonstrated that AS@cRGD‐EVs markedly inhibited the phosphorylation of DRP1 at serine 616 (*p <* 0.05, Figure 7E,F).

### 3.8 Efficacy of Engineered AS@cRGD‐EVs on Mouse RSM Model

**FIGURE 8:**
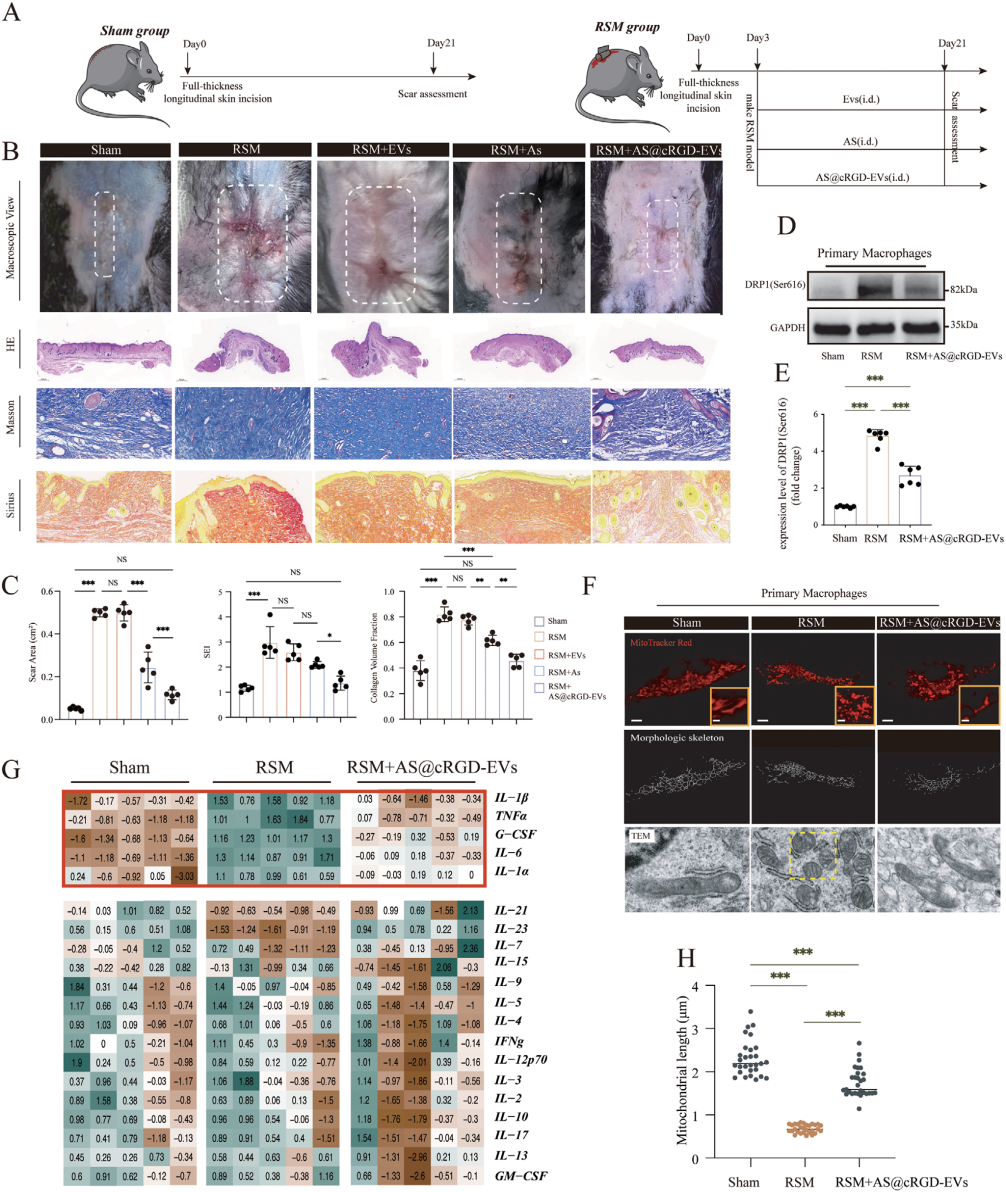
Efficacy of engineered AS@cRGD‐EVs on mouse RSM model. (A) Schematic diagram of studies in animals. (B) Macroscopic photos of mouse scars on post‐injury day 21, scale bar = 1.5 cm; HE staining of newly formed granulation tissue on post‐injury day 21 bar = 500 µm; Masson staining of newly formed granulation tissue on post‐injury day 21, scale bar = 50 µm; Sirus red staining of newly formed granulation tissue on post‐injury day 21, scale bar = 50 µm. (C) Quantitative analysis of surface area of the scars, scar elevation index, and collagen density of the scars, *n* = 5. (D,E) Western blot analysis of DRP1(Ser616) expression level of primary macrophages within different tissues, *n* = 6. (F) The images and morphology analysis of mitochondria via immunofluorescence and TEM, scale bar = 5 µm, with insets providing a higher magnification view, scale bar = 1 µm. (H) The quantitative analysis of mitochondrial length. (G) Heatmap of inflammation array on wound tissues under different conditions. The red boxes indicate pro‐inflammatory factors. (*p < 0.05; ** *p* < 0.01; *** *p* < 0.001. NS = not significant).

Based on the in vitro findings, we further explored the effect of AS@cRGD‐EVs on inhibiting scar formation in vivo. Previously reported mouse scar model RSM was used to evaluate the therapeutic efficacy of AS@cRGD‐EVs. As illustrated in the timeline schematic in Figure 8A, mice were randomly divided into five groups: the Sham group, which received neither RSM modeling nor treatment; the RSM group, which underwent RSM modeling but no treatment; and the remaining three groups received daily intradermal injections of EVs, free AS, or AS@cRGD‐EVs, respectively, following RSM induction. And comprehensive scar assessments were conducted at 21 d for each group. The results demonstrated that AS@cRGD‐EVs effectively improved scar appearance and significantly reduced scar surface area, exhibiting a better anti‐scarring effect compared to free AS (*p <* 0.001, Figure 8B,C). Further histological analysis of the scars revealed that AS@cRGD‐EVs more significantly decreased the SEI (*p <* 0.05, Figure 8B,C) and collagen volume fraction (*p <* 0.01, Figure 8B,C) compared to free AS. These findings confirm that AS@cRGD‐EVs can effectively inhibit scar formation in the mouse RSM model. Further, macrophages in scar tissue were isolated and collected via MACS. Immunofluorescence (IF) assays showed that mitochondrial fragmentation in the RSM + AS@cRGD‐EVs group was significantly reduced compared to that in the RSM group (*p* < 0.001, Figure 8F,H). Concurrently, TEM results showed that macrophages in the scar tissue of the RSM group exhibited shorter mitochondrial lengths and an increased number of fragments, whereas AS@cRGD‐EVs treatment significantly alleviated mitochondrial fragmentation (Figure 8F). Western blot analysis indicated that the expression level of phosphorylated DRP1 (Ser616) in the RSM group was significantly higher than that in the Sham group (*p* < 0.001, Figure 8D,E), while AS@cRGD‐EVs markedly reversed this upregulation (*p* < 0.001, Figure 8D,E). Inflammation assays demonstrated that AS@cRGD‐EVs effectively inhibited the levels of pro‐inflammatory cytokines, including IL‐1β, TNF‐α, G‐CSF, IL‐6, and IL‐1α (Figure 8G).

## 4 Discussion

This study identified excessive mitochondrial fission in macrophages as a critical pathological feature of HS formation, both in human HS tissues and the mouse RSM model. Further exploration found that abnormal mitochondrial dynamics is driven by AURKB‐mediated phosphorylation of DRP1 at ser616, which promotes DRP1 translocation to mitochondria and exacerbates fission. Consequently, this process induces mitochondrial dysfunction and triggers a pro‐inflammatory cascade, which are key mediators of HS progression.

In this study, the mouse RSM model was selected over the established rabbit ear model to address the specific mechanistic aims of this study. Although the rabbit model excels in simulating prolonged inflammation and collagen remodeling seen in human HS [46], the RSM offers two critical advantages for our work: it applies controlled tension to sutured incisions—clinically relevant to tension‑driven scarring—and permits the use of sophisticated murine genetic and molecular tools essential for in‑depth mechanistic dissection (e.g., RNA‑seq, CRISPR‑Cas9, Co‑IP). Our prior validation also showed the RSM reliably induces key HS phenotypes, including macrophage infiltration [19], providing a relevant platform for studying mitochondrial dynamics. Our results align with the growing recognition that mitochondrial dynamics play a pivotal role in inflammation and fibrosis. For instance, studies in fibrosis models have demonstrated that DRP1 inhibition can effectively reverse collagen deposition [47]. Studies also have demonstrated that imbalance in mitochondrial fission/fusion leads to mitochondrial DNA (mtDNA) leakage, which can trigger TLR9/NLRP3 inflammasome activation—a shared molecular mechanism underlying hepatic, pulmonary, and renal fibrosis [48]. These results were echoed in our observation of ROS and mtDNA‐mediated cytokine release in HS macrophages. A key innovation of this study lies in identifying the AURKB‐DRP1(Ser616) axis as a critical regulator of mitochondrial fission in HS macrophages. While DRP1‐mediated mitochondrial fission has been extensively studied, previous investigations have primarily focused on kinases such as CDK1 or CaMKII in DRP1 activation [49]. Here, we provide the first evidence that AURKB directly interacts with DRP1, phosphorylates it at Ser616, and drives excessive fission in HS macrophages—expanding our understanding of post‐translational DRP1 regulation in HS and highlighting a previously unrecognized therapeutic target. Importantly, the phosphorylation‐dependent nature of this regulation suggests a dynamic enzyme‐substrate relationship [50], which may not be fully captured by traditional binary binding assays. Future studies employing phospho‐specific DRP1 mutants and direct kinase assays will be essential to precisely define this mechanism and its therapeutic potential.

The discovery of this mechanism suggests that developing safe and effective drugs targeting the upstream kinase AURKB represents a promising new anti‐scarring strategy. However, existing AURKB inhibitors like Barasertib, which was originally designed to inhibit abnormal tumor cell proliferation through AURKB blockade [51]. But in clinical studies, the use of Barasertib was limited by frequent bone marrow toxicities and poor clinical response [52]. Therefore, there is an urgent need to develop new inhibitors that can simultaneously achieve efficient AURKB inhibition, low toxicity, and suitability for local delivery. Through a comprehensive approach combining machine learning‐based virtual screening of the ZINC20 database with experimental validation, we have demonstrated that AS specifically binds to and inhibits AURKB activity. Structural analyses revealed that AS shares partial binding sites with ATP at Asn‐205 site, suggesting a competitive inhibition mechanism with a dissociation constant (*K*_d_) of 4.393 µm—confirming stable interaction with AURKB. AS has been extensively used in traditional medicine for wound healing and anti‐inflammatory purposes [33]. Unlike the strong cytotoxicity of Barasertib, AS's natural origin endows it with a distinct advantage, CCK‐8 assays in our study showed no cytotoxicity even at concentrations up to 1 mm, supporting its safety profile. Our study firstly demonstrated AS's therapeutic efficacy in HS specifically stems from AURKB inhibition and subsequent suppression of the AURKB‐DRP1(Ser616) axis. This precise mechanistic understanding establishes a solid theoretical foundation for repositioning AS a targeted therapy.

The therapeutic application of AS has been limited by its poor bioavailability, often requiring high or frequent dosing [35]. While existing strategies like nanoparticle encapsulation or microneedle delivery aim to improve solubility and permeability [53, 54], they lack cell‐specific targeting. Here, we developed AS@cRGD‐EVs, which combine the biocompatibility of natural EVs with active targeting via cRGD peptides to integrin αvβ3 overexpressed on macrophages [55]. This design ensures preferential cellular uptake, amplifies AS action on the AURKB‐DRP1 axis within target cells, and minimizes off‑target effects on neighboring wound‑healing processes. While AS@cRGD‐EVs exhibit superior targeting capability and therapeutic efficacy at the wound site, a comprehensive safety assessment of this delivery strategy remains imperative, delineating both the boundaries and future directions of our investigation. First, although intradermal administration aims to maximize local drug concentration, it must be acknowledged that EVs under 200 nm are known to drain from the interstitial space into systemic circulation via lymphatic capillaries, an established physiological clearance route [56]. Consequently, while local delivery may reduce initial systemic exposure compared to intravenous injection, it does not preclude systemic dissemination altogether. A notable limitation of this study is the absence of quantitative biodistribution data for AS@cRGD‐EVs across major organs following intradermal injection. Future studies should employ techniques such as in vivo imaging or isotopic labeling to compare pharmacokinetic profiles across administration routes, thereby precisely delineating local retention versus systemic distribution.

Second, although the cRGD peptide is engineered to selectively bind macrophages overexpressing integrin αvβ3 in wounds, this integrin is also expressed in other pathophysiological contexts, including activated endothelium in tumor vasculature and atherosclerotic plaques [57], as well as in specific cell populations in organs such as the spleen and lungs [58]. Should a fraction of EVs enter systemic circulation, the surface‐conjugated cRGD may actively redirect them to such off‐target sites rather than confining them locally. This ligand‐driven off‐target accumulation represents a fundamental translational challenge for targeted delivery systems. Although no acute systemic toxicity was observed in this study, potential long‐term or subtle biological effects of off‐target accumulation cannot be ruled out. This work establishes a promising proof‐of‐concept for EVs‐mediated targeted therapy. To advance toward clinical translation, subsequent studies must systematically evaluate systemic biodistribution, long‐term retention, and cRGD‐mediated off‐target effects in larger preclinical models. Defining these parameters will be critical for establishing the therapeutic window and safety profile of this approach.

Beyond HS, these findings highlight the broader potential of targeting mitochondrial dynamics in inflammation‐driven fibrosis, with the AURKB‐DRP1(Ser616) axis emerging as a conserved therapeutic node. Ultimately, this work underscores the value of integrating mechanistic insights with innovative delivery strategies to bridge the gap between basic research and clinical application, offering hope for more effective, precise treatments for HS and beyond.

## 5 Conclusions

This study identifies a key pathomechanism in HS formation, centered on the AURKB‐DRP1(Ser616) axis‐driven excessive mitochondrial fission in wound macrophages. To therapeutically intervene, we engineered AS@cRGD‐EVs, a novel nanotherapeutic that utilizes cRGD‐mediated targeting for the specific delivery of the AURKB inhibitor AS to regulate macrophages, thereby overcoming its intrinsic delivery limitations. Treatment with AS@cRGD‐EVs effectively restored physiological mitochondrial dynamics, suppressed pathological inflammation, and attenuated fibrotic outcomes in vivo. These findings not only establish a new mitochondrial‐centric paradigm in HS pathogenesis but also present a translatable, cell‐targeted therapeutic strategy with broader potential for treating macrophage‐driven fibrotic diseases.

## Author Contributions

L.Y.L., Y.Z., and Z.Z. conceived and designed the experiments. L.Y.L. and C.L.S. performed all the experiments. L.Y.L., S.F.G., and Y.S. analyzed the data. L.Y.L., X.J.W., and X.W. prepared the figures. L.Y.L., X.W., X.J.W., and Y.S. contributed the reagents/materials/analysis tools. L.Y.L. and Y.W. wrote the paper. Y.W. revised the manuscript. All authors have read and approved the final manuscript.

## Funding

This work was supported by the Natural Science Foundation of Shanghai Project (grant no.19ZR1430200).

## Ethics Statement

Male C57BL/6 mice, aged six to eight weeks, were obtained from LingChang Biomedical Ltd, (Shanghai, China). The research involving animals strictly adhered to ethical standards and protocols and was conducted with approval from the ethical review board of Shanghai Ninth People's Hospital, Shanghai Jiao Tong University School of Medicine, Shanghai, China (SH9H‐2020‐A314‐1, March 14, 2020). Every effort was made to minimize the use of experimental animals and to mitigate any potential suffering they might experience. HS tissue samples obtained from patients who had undergone HS excision surgery. Ethical approval for the collection of clinical samples involving human subjects was granted by the ethical review board of Shanghai Ninth People's Hospital, Shanghai JiaoTong University School of Medicine, Shanghai, China (SH9H‐2024‐TK400‐1, February, 28, 2024).

## Conflicts of Interest

The authors declare no conflicts of interest.

## Supporting information




**Supporting File**: advs74258‐sup‐0001‐SuppMat.docx.

## Data Availability

The data that support the findings of this study are available from the corresponding author upon reasonable request.
