# Supplementary material for: Targeted Extracellular Vesicles Deliver Asiaticoside to Inhibit AURKB/DRP1‐Mediated Mitochondrial Fission and Attenuate Hypertrophic Scar Formation
Source: Adv Sci (Weinh). 2026 Feb 4;13(21):e17108. doi: 10.1002/advs.202517108 (PMC13073238; doi:10.1002/advs.202517108)
Supplement: Supplementary file 1 — Supporting File: advs74258‐sup‐0001‐SuppMat.docx. [file ADVS-13-e17108-s001.docx]

**Supporting Information**

**
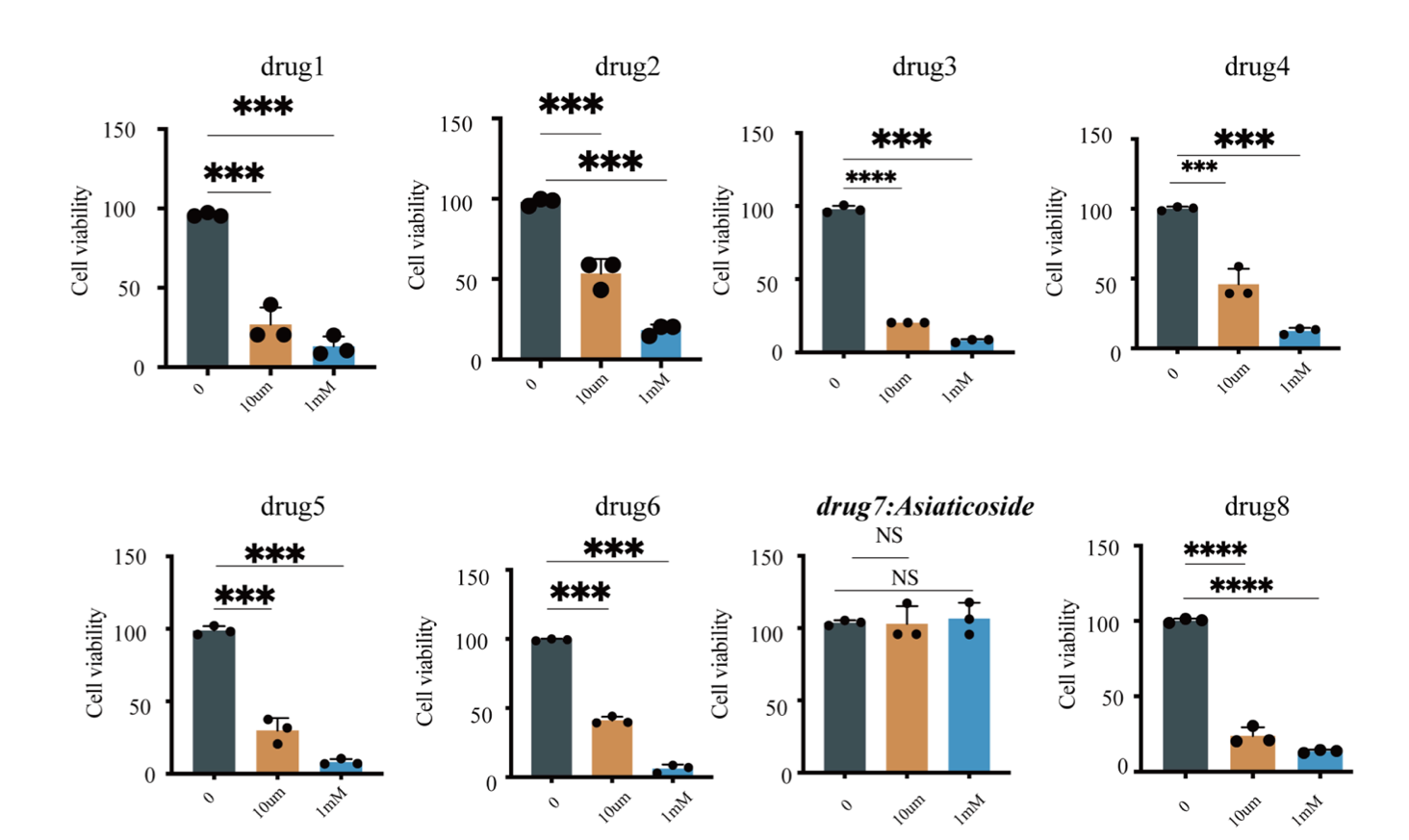
Figure S1.** Cell compatibility of drugs target to AURKB.


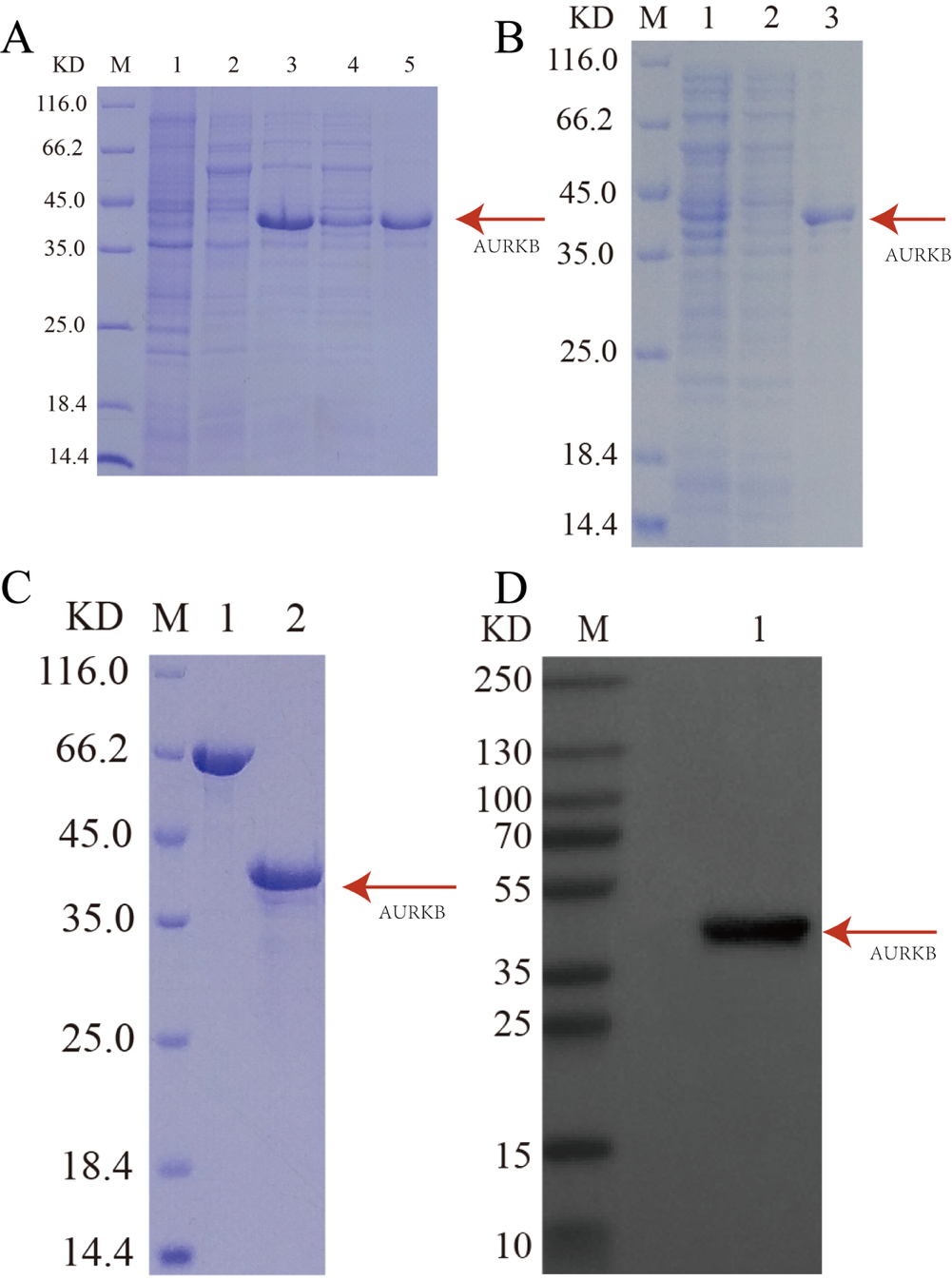
**Figure S2.** Identification and Purification Analysis of AURKB. A) Protein expression identification SDS-PAGE analysis. M: Protein molecular weight standard. 1: PCZN1 induction (empty load). 2: Not induced. 3: After induction. 4: Induced fragmentation of supernatant. 5: Induced precipitation after fragmentation. B) Protein Purification SDS-PAGE Analysis. M: Protein molecular weight standard. 1: Processed samples after crushing. 2: Outflow. 3: Wash out. C) Protein quality inspection analysis. M: Protein molecular weight standard. 1：0.5 mg/mL BSA. 2: Purified sample. D) Protein Western Blot Identification and Analysis. M: Protein molecular weight standard. 1: Purified sample.

**Figure S3.** Raw data of Western blot experiments.

**
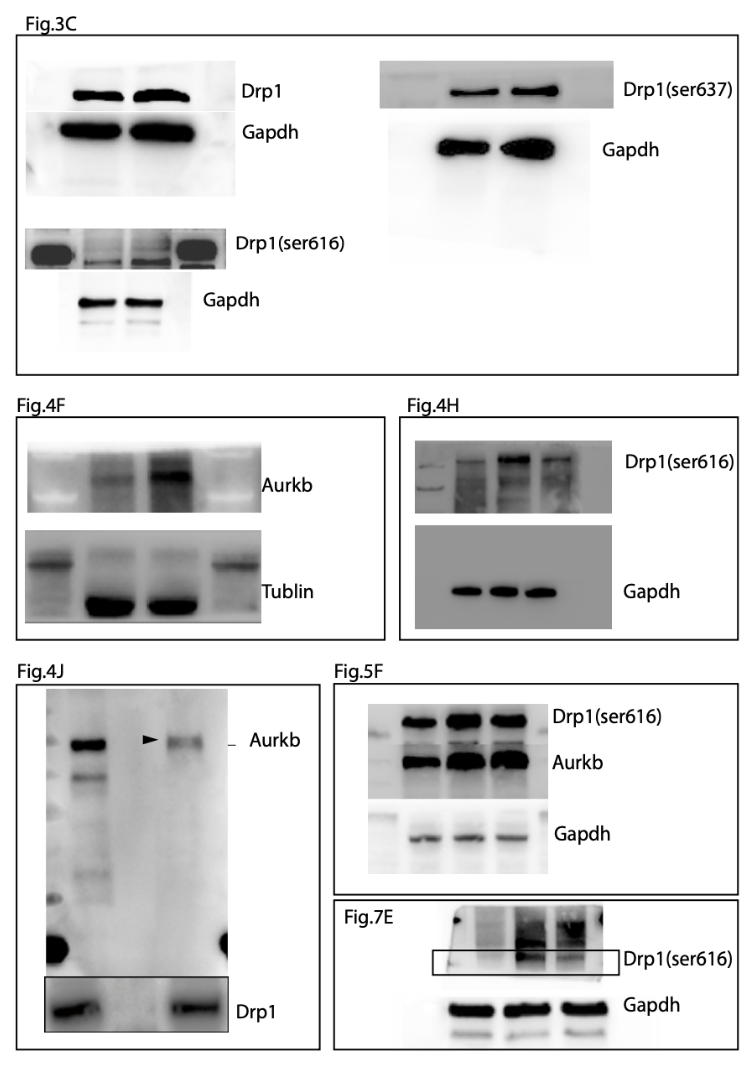
**


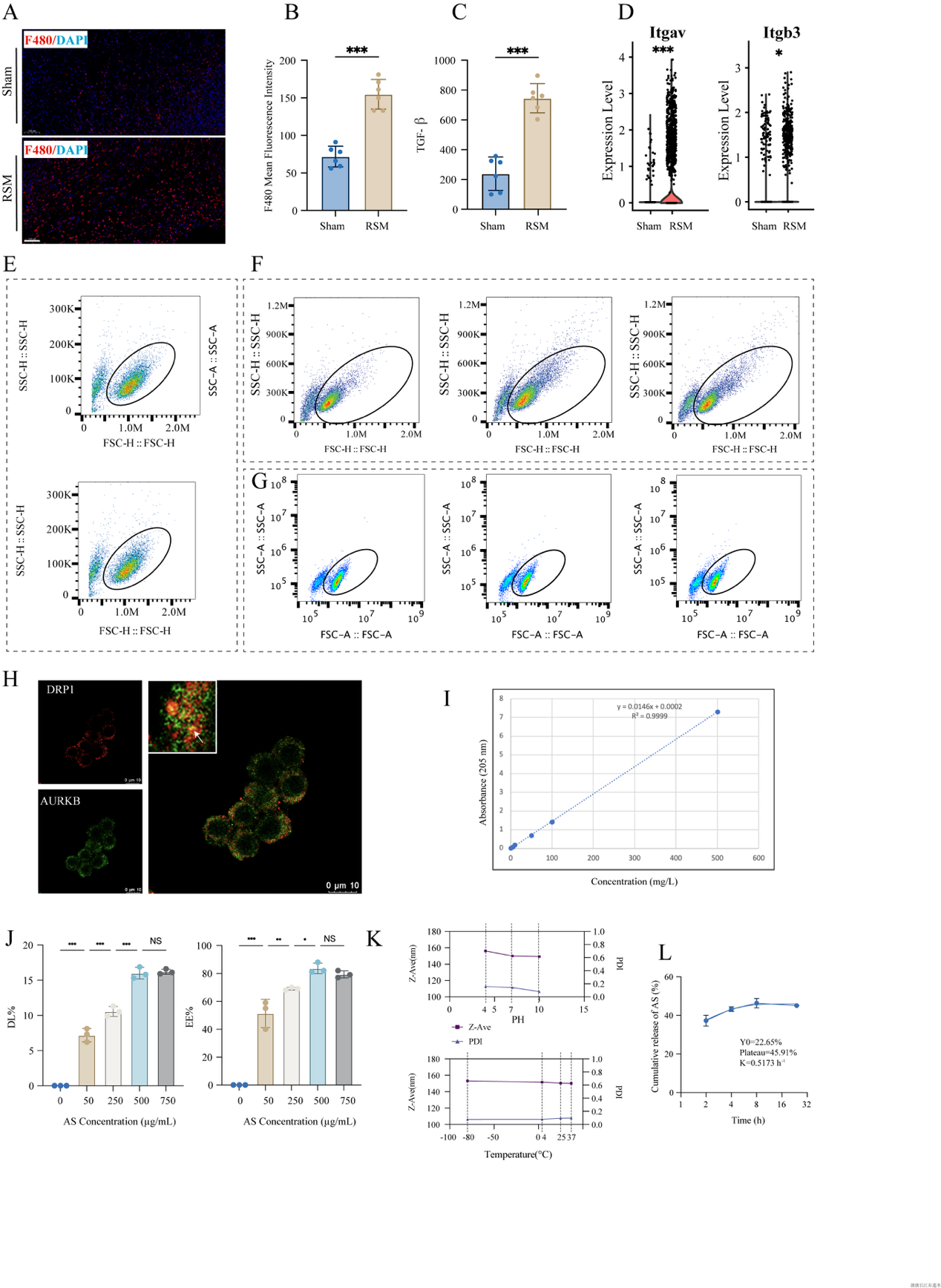
**Figure S4.** (A-B) Representative immunofluorescence images and semi-quantitative analysis of F4/80⁺ macrophages in wound tissues from Sham and RSM groups. (C) ELISA quantification of TGF-β levels in tissue homogenate supernatants from Sham and RSM wounds. (D) Violin plots showing expression levels of Itgav and Itgb3 in macrophages from single‑cell sequencing data of Sham and RSM tissues. (E-G) Representative gating strategies used in flow cytometry analysis. (H) Immunofluorescence co‑localization of AURKB and DRP1 in Raw264.7 cells; arrows indicate sites of protein co‑localization. (I) Standard curve of asiaticoside (AS) concentration versus absorbance at 205 nm. (J) Effects of different AS concentrations on exosome encapsulation efficiency and drug loading capacity. (K) Stability assessment of AS@cRGD‑EVs under varying pH and temperature conditions, measured by particle size and PDI. (L) In vitro release kinetics profile of AS from AS@cRGD‑EVs. (**P <* 0.05; ** *P* < 0.01; *** *P* < 0.001. NS= not significant).

**Figure S5.** A schematic diagram illustrating the architecture and data flow of the MPNN-CNN model.

**
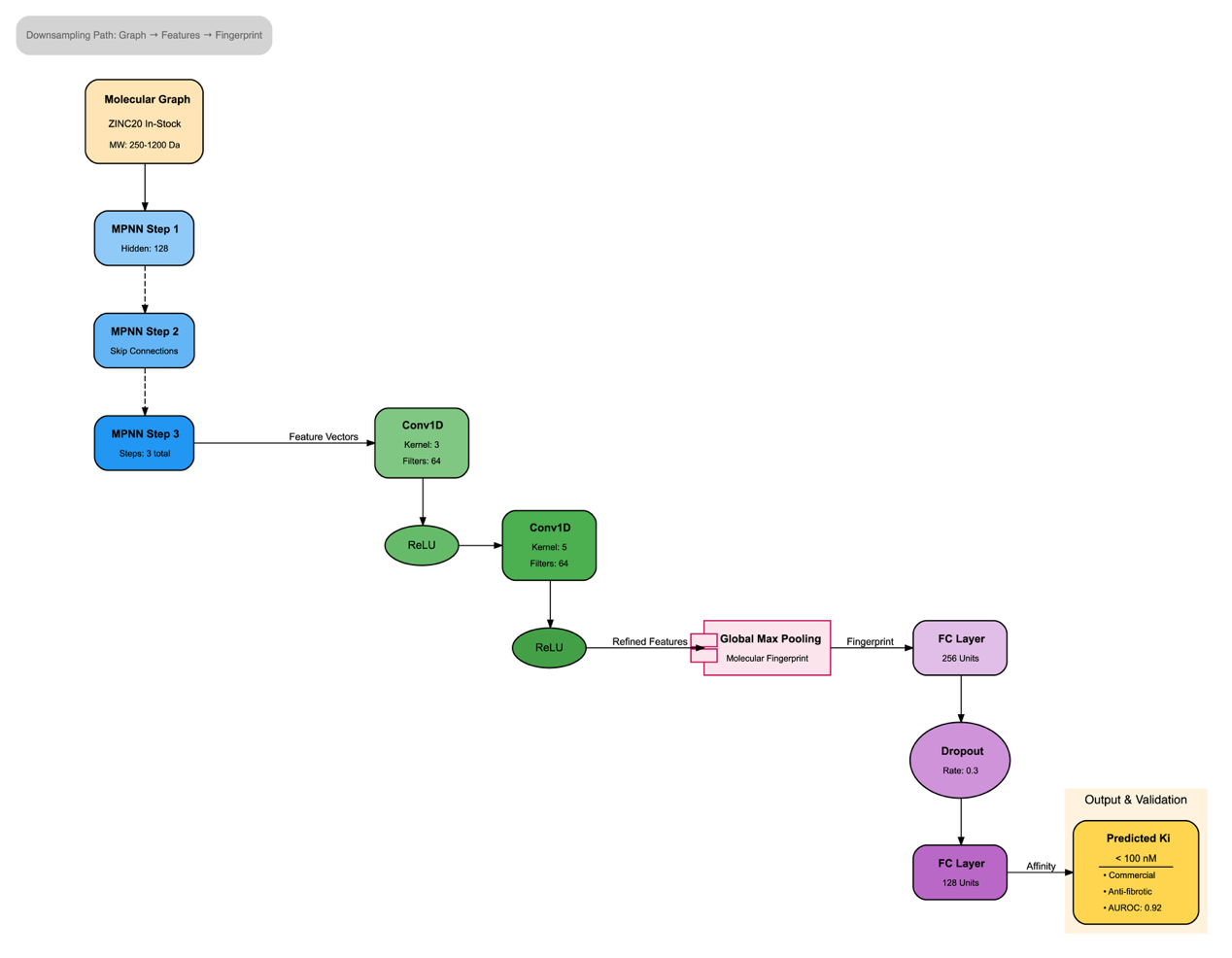
**

**Table S1.** Information of drugs target to AURKB.

| Drug information | | |
| --- | --- | --- |
| Compound CID | SMILES | Predicted Ki (nM) |
| Drug1: 1150573 | CC1=CC=C(C=C1)N2C(=NN=C2SCC(=O)OC3CCCCC3)C4=CC=NC=C4 | 2.75E-10 |
| Drug2: 1425856 | C1=CC=C(C(=C1)CN2C3=CC=CC=C3[C@@](C2=O)(CC(=O)C4=CC=CC=C4O)O)F | 2.70E-05 |
| Drug3: 2569575 | CC1=CC2=NN=C(N2C3=C1C=CC(=C3)OC)SCC(=O)N[C@@](C)(C#N)C4CC4 | 0.001078167 |
| Drug4: 25600120 | CC1=C(C(=NN1C)C)NC(=O)/C=C/C2=CC(=C(C=C2)OCC=C)OC | 1.856222479 |
| Drug5: 95232323 | CC1=CC2=NN=C(N2C(=N1)C)S[C@H](C)C(=O)C3=C(C=C(C=C3)F)F | 5.348704391 |
| Drug6: 16625763 | COC1=CC(=CC(=C1)NC(=O)C2=CC3=CC=CC=C3C=C2NS(=O)(=O)C4=CC=CS4)OC | 16.11943359 |
| Drug7: 11954171 | C[C@@H]1CC[C@@]2(CC[C@@]3(C(=CC[C@H]4[C@]3(CC[C@@H]5[C@@]4(C[C@H]([C@@H]([C@@]5(C)CO)O)O)C)C)[C@@H]2[C@H]1C)C)C(=O)O[C@H]6[C@@H]([C@H]([C@@H]([C@H](O6)CO[C@H]7[C@@H]([C@H]([C@@H]([C@H](O7)CO)O[C@H]8[C@@H]([C@@H]([C@H]([C@@H](O8)C)O)O)O)O)O)O)O)O | 68.41174867 |
| Drug8: 17486683 | CCN1C2=CC=CC=C2N(C1=O)CC3=NN=C(O3)C4=CC=C(C=C4)OC | 93.77239008 |

| Time | 1 | 2 | 3 | 4 | 5 | 6 | 7 | 8 | 9 |
| --- | --- | --- | --- | --- | --- | --- | --- | --- | --- |
| Affinity(kcal/mol) | -8.4 | -8.4 | -7.9 | -7.8 | -7.5 | -7.4 | -7.4 | -7.3 | -7.3 |
| Calculations | mean ± SD =-7.71± 0.44 kcal/mol | | | | | | | | |

**Table S2.** The docking affinity of AC and AURKB.

**Table S3.** The docking affinity of ATP and AURKB

| Time | 1 | 2 | 3 | 4 | 5 | 6 | 7 | 8 | 9 |
| --- | --- | --- | --- | --- | --- | --- | --- | --- | --- |
| Affinity(kcal/mol) | -7.2 | -6.7 | -6.5 | -6.5 | -6.5 | -6.5 | -6.4 | -6.4 | -6.4 |
| Calculations | mean ± SD =-6.57± 0.25 kcal/mol | | | | | | | | |

**Table S4. In vitro synthesis of AURKB gene using plasmid as a vector.**

| **Restriction Digestion Map** | | | | |
| --- | --- | --- | --- | --- |
|  | **1** | **2** | **M** |  |
| 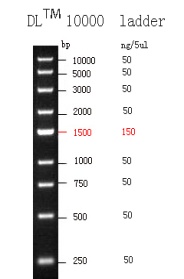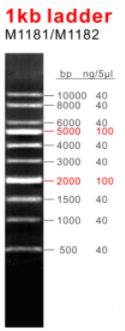 | 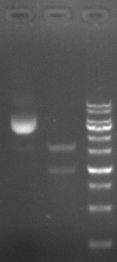 | | | **Lane 1：**Plasmid  **Lane 2:** Plasmid Digested with XbaI and ApaI  **Lane M**: DNA Marker |
